# Fermentative Foods: Microbiology, Biochemistry, Potential Human Health Benefits and Public Health Issues

**DOI:** 10.3390/foods10010069

**Published:** 2020-12-31

**Authors:** Chrysa Voidarou, Maria Antoniadou, Georgios Rozos, Athina Tzora, Ioannis Skoufos, Theodoros Varzakas, Areti Lagiou, Eugenia Bezirtzoglou

**Affiliations:** 1 Laboratory of Animal Health, Food Hygiene and Quality, Department of Agriculture, School of Agriculture, University of Ioannina, 47132 Arta, Greece; xvoidarou@uoi.gr (C.V.); tzora@uoi.gr (A.T.); jskoufos@uoi.gr (I.S.); 2 School of Dentistry, National and Kapodistrian University of Athens, 11521 Athens, Greece; mantonia@dent.uoa.gr; 3 Laboratory of Microbiology, Biotechnology & Hygiene, Department of Agricultural Development, Democritus University of Thrace, 68200 Orestiada, Greece; clevervet@hotmail.com; 4 Department of Food Science and Technology, University of the Peloponnese, 24100 Kalamata, Greece; 5 Department of Public and Community Health, University of West Attika, 11521 Athens, Greece; alagiou@uniwa.gr; 6 Laboratory of Hygiene and Environmental Protection, Medical School, Democritus University of Thrace, 68100 Alexandroupolis, Greece; empezirt@yahoo.gr

**Keywords:** fermented foods, microbiology, microbiome, health, oral health

## Abstract

Fermented foods identify cultures and civilizations. History, climate and the particulars of local production of raw materials have urged humanity to exploit various pathways of fermentation to produce a wide variety of traditional edible products which represent adaptations to specific conditions. Nowadays, industrial-scale production has flooded the markets with ferments. According to recent estimates, the current size of the global market of fermented foods is in the vicinity of USD 30 billion, with increasing trends. Modern challenges include tailor-made fermented foods for people with special dietary needs, such as patients suffering from Crohn’s disease or other ailments. Another major challenge concerns the safety of artisan fermented products, an issue that could be tackled with the aid of molecular biology and concerns not only the presence of pathogens but also the foodborne microbial resistance. The basis of all these is, of course, the microbiome, an aggregation of different species of bacteria and yeasts that thrives on the carbohydrates of the raw materials. In this review, the microbiology of fermented foods is discussed with a special reference to groups of products and to specific products indicative of the diversity that a fermentation process can take. Their impact is also discussed with emphasis on health and oral health status. From Hippocrates until modern approaches to disease therapy, diet was thought to be of the most important factors for health stability of the human natural microbiome. After all, to quote Pasteur, “Gentlemen, the microbes will have the last word for human health.” In that sense, it is the microbiomes of fermented foods that will acquire a leading role in future nutrition and therapeutics.

## 1. Introduction

Louis Pasteur discussed the issue of fermentation [1,2]. He obviously suspected—and, in some cases, he knew—that fermentative processes are diverse and complicated. Indeed, although modern science recognizes more than one type of fermentation, a general definition should define fermentation as a biochemical process through which most microorganisms decompose carbohydrates to produce energy under anaerobic conditions [3,4,5]. According to Pasteur, “Fermentation is life in the absence of oxygen” [1]. In this somewhat obscure and phenomenological term, various types of fermentation are included, such as yeast fermentation, lactic acid fermentation, butyric acid fermentation, propionic acid fermentation and acetic acid fermentation. The diversity of the phenomenon leads to a variety of end products, which include CO_2,_ ethanol, organic acids and other organic molecules. It is many of these products that make fermentation so useful and interesting both to science and industry [3,4,5,6,7].

**Figure 1:**
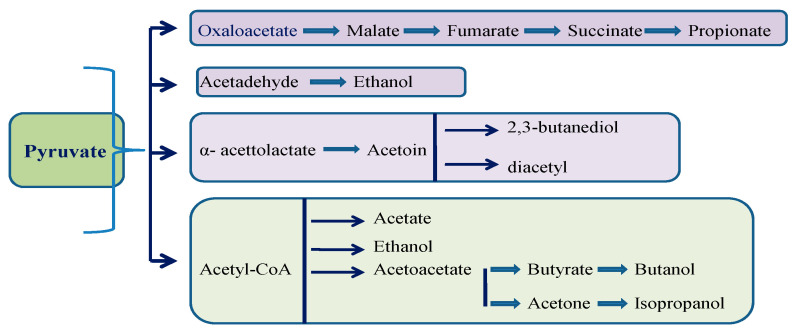
The diversity of fermentations. One molecule of glucose through glycolysis results in two molecules of pyruvate. Under anaerobic conditions and depending on the microorganism involved, fermentation can continue in certain pathways.

The process of carbohydrate decomposition creates a flow of protons and electrons. In aerobic conditions, the final receptor is oxygen (respiration), andin anaerobic conditions, the final receptors are other organic molecules, such as pyruvate or acetyl CoA (fermentation). Fermentation yields much less energy than respiration. The progressive oxidation of sugars involves the transfer of hydrogen ions from intermediate products in the pathway to the final receptor. The Embden–Meyerhof–Parnas pathway (EMP) is utilized in most cases to decompose glucose to pyruvate (homofermentive strains). The Entner–Doudoroff pathway decomposes lactose molecules, while the heterofermentive strains decompose pentoses and other sugars through the phosphoacetylase pathway. The end products depend on the substrate as well as the microorganism species and strain (Figure 1 and Table 1). Different species and different genera have different capacities of fermentation [3,6,7]. For example, *E. coli* has limited fermentation capacities regarding the variety of end products as compared to lactic acid bacteria (LAB). On the other hand, beneficial fermentation properties can be engineered. By inserting a lactic acid dehydrogenase gene from a bovine muscle, *Saccharomyces cerevisiae* can produce lactic acid in levels similar tothose of LAB [8].

Microorganisms and carbohydrates are abundant in nature and fermentation occurs in every anaerobic environment. It follows that it is not a question of “if’ but a question of “when” and “how” early humans discovered and harvested the benefits of fermentations in the domain of food and beverage. Near Lake Neuchatel in Switzerland, archaeologists discovered the remains of cheese manufacturing from 6000 BCE [9]. Homer refers to Cyclops Polyphemus as a cheese producer, while in many cultures, such as the Mongols, the Tuareg, the Bedouin, the Tibetans many central Africa tribes and Amazon natives, among others, strong evidence exists of fermented curds production in the antiquity [10,11]. Bread is depicted in ancient Egypt scrolls aging from 2700–2500 BCE. In the third century CE, 72 kinds of bread were listed in Athens [11]. Finally, wine must have been “invented” somewhere between the Black Sea and the Caucasus mountains from 6000 to 3500 BCE. Apparently, someone accidentally left crashed grapes in a container and tasted them much later [12,13,14]. A similar theory is presented for beer brewery, but in this case, meshed cereals with water were left the container [15]. In ancient Egypt, the kneading and baking facilities were beside the beer breweries, obviously both using *Saccharomyces cerevisiae* [15,16].

Since the first attempts of ancient cultures to empirically exploit the fermentation phenomena, solid traditions have been established, resulting in very wide variety of fermented foods and beverages globally [4,15]. Even today, such products are in demand and highly valued in every food market. Yet, the local traditional produce is limited and faces various safety issues (particularly for dairy products and fermented meats) [17]. The food industry uses different fermentation techniques than artisan producers. Instead of the so-called “wild” or “spontaneous” ferments, a mainstream traditional method, industrial-scale fermentation, is achieved by the addition of specific “starter” cultures which initiate the fermentation process [15,18,19]. For some products secondary cultures must be added in the process. Industrial ferments have been criticized for having a “flat” taste in comparison to the rich palate of their artisan counterparts. The reason for that is the obvious one: For their fermentation, artisan products rely on a wide variety of microorganisms originating from the local environment, while industrial products are fermented by artificial cultures of a significantly smaller number of bacteria and fungi. Rival critics judge the artisan products not only for their dubious safety but also for the fluctuation of their sensorial characteristics, which vary depending on numerous factors such as the environment, the microclimate, and many others [20,21,22].

This debate is fuelled by the behaviour and characteristics of the microbiome responsible for the fermentations, “wild” or industrial.

The fact is that value is added to foods through fermentation because they (i) contribute to their preservation; (ii) actively participate in the development of their texture, flavour and aroma; (iii) help to eliminate pathogens and toxic substances; (iv) improve digestibility; (v) create new products for new markets and (vi) increase dietary value [21,22,23].

It is not a coincidence that most probiotic strains have originated from microbiomes of fermentations. These microbiomes have been proved safe and beneficial throughout history. The positive impact of foods containing probiotics to human health has been extensively discussed in the scientific literature and it is a proven fact that probiotics contribute positively to the control and treatment of inflammatory bowel diseases [24] and of various gastric and enteric diseases [24,25,26], to the alleviation of patients suffering from Crohn’s disease [27], to the control of certain allergies [28], to the prevention and treatment of infections of the urinary tract [29,30] and to the control and reduction of high blood cholesterol levels [31]. Many special diets and, of course, most functional foods contain such microorganisms, not to mention the supplements. The global probiotics market is projected to reach USD 77 billion by 2025 and some future trends have already been shaped, such as tailored and personalized probiotics based on an individual’s own flora for increased colonization of the digestive tract [32].

The aim of this review is to discuss and describe certain issues regarding the fermented foods, namely their microbiology, their variety and their safety with respect to their microbiome. The authors opted for a special reference to the impact of these products to oral health not only as an indicative issue to demonstrate their general impact to human health, but also because the particular impact to oral health, although not a less important aspect, has seen much less publicity.

## 2. Industrial and Artisan Fermentations

### 2.1. Microbiology of Fermented Foods

The process of fermentation, along with the processes of drying, smoking and salting, is one of the oldest methods of food and beverage preservation and of improving their nutritional value. Fermentation is a biotechnology that promotes and controls the growth of microorganisms and their metabolic activities for the preservation and transformation of raw food materials [33,34]. Therefore, today, when one considers fermented foods, it is not surprising that the focus is on foods in which microbial activity plays an essential role in obtaining the required stability, safety and sensory properties [35]. This discussion excludes those products that are often described as fermented but are largely the product of non microbial, enzymatic processes, such as black tea and Southeast Asian fish sauces, but should include those products such as tempeh and vinegar, in which the main microbial activity is not that of fermenters [36]. However, when the discussion comes to the virtues of fermentation as a preservative, it is almost always related to those foods where lactic acid bacteria (LAB) play a central role in the production process [37,38,39].

During food fermentation, metabolites produced by the desirable fermenting organisms, such as lactic acid, acetic acid, carbon dioxide, ethanol, hydrogen peroxide, bacteriocins and antimicrobial peptides, acting alone or in combination, inhibit the growth of spoilage and pathogenic organisms, there by achieving an extension in the shelf life of susceptible products [15].

The fermentation of foods and beverages, at the level of know-how, has a past of at least 6000 years and is likely to be the result of natural and spontaneous microbial interactions [40,41]. Today, food fermentation can be achieved through two main production methods. With the first production method, the fermentation process is driven by the autochthonous (native/indigenous) microflora of the raw food materials or processing environment, for instance, sauerkraut, kimchi and certain fermented soy products [20,37,42,43,44]. These types of fermented foods are often named by researchers, or food technologists, as “wild ferments” or “spontaneous ferments” [20,42,43,44,45,46,47]. With the second method, to achieve fermentation, it is necessary to add starter cultures, known as “culture-dependent ferments,” for example, kefir, yoghurt, kombucha and natto [20,22]. One method of performing a culture-dependent ferment is “backslopping,” in which a small amount of a previously fermented batch is added to the raw food, for example, sourdough bread [20,47,48]. Starters used to initiate fermentation can be either natural (e.g., backslopping), or selected commercial starters to standardize the organoleptic characteristics of the final product [20,48]. Compiling a geographical list, we can place Europe, North America, Australia and New Zealand in the areas where fermented products are produced mainly using defined starter cultures, while Asia and Africa are extraordinary ambassadors of spontaneous fermentation [40,49].

Moreover, traditional food fermentation processes can be broadly classified into (a) lactic acid fermentation, (b) fungal fermentation and (c) alkaline fermentation [15,19,49,50,51]. Examples of lactic acid fermented products, i.e., products primarily fermented by lactic acid bacteria, include yoghurt, sausages, cheese, sauerkraut (fermented cabbage from eastern and central Europe), kimchi (fermented and spiced Napa cabbage from Korea) [15,19] and soy sauce (ganjang) [52]. Yeasts are also involved in the fermentation of many of the lactic acid-fermented products, including kefir (a slightly alcoholic dairy beverage from the Caucasus) and kombucha (a fermented sweetened tea from China) [51,53]. Most of the well-known soy-based fermented foods from Asia, such as tempeh and soy sauce, are produced by fungal fermentation, except natto, which is produced by alkaline fermentation [54,55]. Industrial fermentation processes use either submerged or solid-state bioreactors that are conducted in batch, semi-batch or continuous mode. Most food fermentation processes, from sauerkraut and kimchi to miso and tempeh, use solid-state fermentation processes conducted in batch mode, where microorganisms are cultivated on the surface of a water-insoluble substrate [20,56]. Submerged fermentation processes are used in the production of yoghurt and other dairy-based beverages, alcoholic beverages and food condiments such as vinegar [36,57].

There are two types of fermentation (regarding the way the fermentation microorganisms are utilized):

(A) Natural fermentation: A fermentation in which the fermentation microorganisms are already part of the natural microflora present in the foodstuff, so they do not need to be added. The only thing required is to create the necessary conditions for their development (e.g., the creation of anaerobic conditions, as in the production of pickles or olives), or the suppression of competing microflora (e.g., with the addition of salt or vinegar to the products) [15,19,58].

(Β) Controlled fermentation with starter cultures: A fermentation that starts with the addition of a suitable inoculation with a large population of the desired fermentation microorganisms, necessary when the raw material is pasteurized (e.g., pasteurized milk) or when it is difficult for the desired fermentation microorganisms to prevail over competing microorganisms (e.g., in beer brewing, where wild yeasts may prevent the alcoholic fermentation by the strains of *Saccharomyces*). The starter culture contains the natural fermentation agents already present in the microflora of the food, but in much higher concentration than normal, to ensure that they easily prevail over competing–spoiling microorganisms. Therefore, with the addition of a starter culture, we better ensure a smooth fermentation process, the prevention of spoilage and the standardization of the product (steady qualitative and organoleptic characteristics) [15,19,59].

Today, fermentation is no longer simply considered a method of food preservation. Through time, man has managed to control the fermentation process, and fermented food now constitutes a separate sector of the food industry [22,40,60]. In Germany, for example, approximately 25% of consumers prefer fermented food [20,61]. As a result of fermentation, the taste, smell, appearance, structure, texture, lifespan and safety of fermented food has different qualities compared to the raw materials from which they are produced [15,19,62]. For example, the reduction of pH in meat paste can be achieved with the addition of Glucono-delta-lactone (GDL) or through fermentation [63]. In both cases, the products are safe for consumers and have the same shelf-life and coherence stability, but the distinctive, desired taste of the final product can only be achieved through fermentation [64,65].

In addition, consumers have been consuming fermented food and beverages for a very long time, being aware that, in the process, they are ingesting billions of living cells of specific microorganisms or/and the metabolic products of these microorganisms without fear for their health and safety [22,65,66]. This fact highlights the resilience of consuming fermented food through time and encourages researchers to continue their never-ending efforts to document the microflora of fermented food and find antimicrobial compounds that may be naturally present in such food [67]. They can then research the possible use of such preserving agents as natural bio-preservatives, instead of chemical preservatives in other food products [68,69,70].

The bibliographical references regarding the microbial ecology of fermented foods and beverages in various regions such as Asia [19,38,40,58], Africa [71,72,73], Europe [74,75,76], South America [21,37,40] and North America [23,77] are numerous and many species of microorganisms have been documented. In the last few years, the use of molecular techniques and tools have helped clarify, at least partially, the confusion present in the naming of newly isolated microorganisms and the generalisation which is caused by the use of conventional classification interpretation of the documented phenotypical characteristics [78,79]. In simple terms, these molecular techniques, such as nucleic acid probe, species-specific polymerase chain reaction (PCR), Rep-PCR, multiplex PCR, 16S rDNA sequencing, DGGE and TTGE, offer reliable solutions for microbial identification in fermented foods and are often used to analyse the microbial flora of fermented food products [78,79,80,81,82,83].

Although some fermented foods contain only few dominant consortia of microorganisms, the dynamic of the microbial population during the fermentation process can develop into an extremely complex phenomenon. In certain foods, even small changes in the variety and number of bacteria can lead to significant differences in the end products compared to the initial planning and to variations in their quality and organoleptic (sensory) properties [20,22]. Therefore, the stability and hardiness of the utilised consortia of microorganisms, in turn, lead to fermentation processes and procedures characterized by their consistency, which guarantees the production of high-quality foodstuffs.

### 2.2. Main Microorganisms in Fermented Foods Ecosystems

#### 2.2.1. Bacteria

Bacteria are the dominant microorganisms not only in foods which have undergone natural fermentation, but also in those which are fermented with the use of starter cultures. Among bacteria, lactic acid bacteria (LAB) are more commonly found in the production of acidic fermented products. Non-LAB bacteria, such as Bacillus, Micrococcaceae, Bifidobacterium, Brachybacterium, Brevibacterium, Propionibacterium, etc., are also involved in food fermentation, mainly as a secondary group of microorganisms used to aid the smooth development of the fermentation process [19,38,84,85,86,87,88,89].

Despite the diversity of the bacteria directly or indirectly involved in the production of fermented foodstuffs, at present, all are classified into one of three Phyla: Firmicutes, Proteobacteria and Actinobacteria [87]. LAB bacteria, which are a complex/compound of Gram-positive bacteria and one of the main microorganisms used in the production of fermented product, can be found within the Firmicutes group (Table 2). It should be emphasized that the Phyla Cyanobacteria are found in fermented butter and a rice cake called Idli, but researches have assumed that the isolated cyanobacterial sequences correspond to cereal chloroplasts [90].

#### 2.2.2. Fungi

Although the sight of mould in food is a sign that it is contaminated and should be discarded, there are some foods in which the presence of visible fungal mycelium is very much a part of the combiota and food dynamics.

Different fungal genera exist, such as Actinomucor, Amylomyces, Aspergillus, Bjerkandera, Brettanomyces, Candida, Cryptococcus, Cyberlindnera, Cystofilobasidium, Debaryomyces, Dekkera, *Fusarium*, Galactomyces, Geotrichum, Cladosporium, Guehomyces, Hanseniaspora, Hansenula, Hyphopichia, Issatchenkia, Kazachstania, Kluyveromyces, Lecanicillium, Lachancea, Metschnikowia, Monascus, Mucor, Neurospora, Penicillium, Pichia, Rhodotorula, Rhodosporidium, Rhizopus, Saccharomyces, Saccharomycodes, Saccharomycopsis, Schizosaccharomyces, Schwanniomyces, Sporobolomyces, Scopulariopsis, Sporendonema, Starmerella, Torulaspora, Trametes, Trichosporon, Trigonopsis, Torulopsis, Ustilago, *Wickerhamomyces*, Yarrowia, Zygosaccharomyces and Zygotorulaspora [12,49,116].

Filamentous fungi: Several species of filamentous fungi become the vehicle of choice to produce essential microbiota for alcohol-producing dry starters, fermented milk and cheeses, and are commonly used in Asia and Europe [19,38,87,117]. In particular, filamentous fungi present in traditional starters from Asia have several functionalities, such as saccharification, liquefaction and ethanol production to produce different types of low-alcoholic beverages and high-alcoholic distilled liquor [117,118,119,120,121]. In Europe, they are used in the development of different dairy products, ripening of various types of cheese (e.g., Roquefort, Camembert) and enzyme production [38,122,123].

Data for some important Fungal genera:

Within the genus Aspergillus, Aspergillus acidus, *A.* oryzae, *A*. sojae, A. oryzae, *A*. niger, *A. sydowii, A. flavus* and *A. versicolor* are used in the production of miso and soya sauce fermentations, sake and awamori liquors and in the fermentation of Puerh tea, respectively [117,124,125,126].

Aspergillus is a phenotypically polythetic genus and is widely distributed in the environment [127], with some of its species having sporulating ability [128]. The latter two abovementioned species are under the light of the scepticism for many researchers. Theybe found in traditionally prepared starters, but it is well known that *A. flavu,* produces aflatoxin and *A. versicolor* toxic metabolites. The question that arises is whether the presence of these specific species is desirable because it imparts specific organoleptic characteristics to the final fermented product, as well as whether the coexistence of other species of filamentous moulds and lactic acid bacteria may play an antagonistic role against Aspergillus species inside the structure of the food matrix, which potentially leads to a reduction of aflatoxin production [129,130,131]. Genus Fusarium currently contains nearly less than 200 accepted species, with the most common in food industry (cheese fermentations), Fusarium domesticum, F. solaniand F. venenatum [132,133]. Species from the *Fusarium* genera are recognized as promising sources of several enzymes for industrial application, as well as “helpers” which promote the production of aromas and pigments to the final products. Some of the secondary metabolites produced by *Fusarium* sp. strains are mycotoxins that affect human and animal health, but studies have demonstrated that the production of mycotoxins stops under industrial conditions [38,134].

Within the genus Rhizopus, Rhizopus oligosporus, *R. microsporus R. stolonifer*, *R. arrhizus, R. delemar, Rhizopus oryzae, R. delemar*. Rhizopus sp. Strains were detected in yaoqu from China and banh men from Vietnam, which are strong amylase producers [117,135,136]. Also used in the fermentation process of tempeh, a fermented soybean food [117,137,138], *R. oryzae* is considered as a GRAS filamentous fungus [139], which is commonly used for production of some Asian fermented foods [19,38,117,135,140].

Within the genus Penicillium, Penicillium camemberti, P. biforme, P. fuscoglaucum, P. palitans, *P.* commune (as the wild-type “ancestor” of *P.* camemberti), P. biforme, P. fuscoglaucum, P. palitans, P. caseifulvum, P. nalgiovense P. chrysogenum, P. commune, P. solitum, P. nordicum andP. expansum are the most common species involved in the microbiotaof several types of cheeses, such as Camembert, Brie, and “blue cheeses,” as well as fermented mild-ripened sausages (like salami) and raw dry- or smoke-cured meat products (like ham) [141,142,143]. All blue-mould cheeses are always made with P. roqueforti, which is inoculated and grows inside the product. P. camemberti is used in mould-ripened soft cheeses such as Camembert and Brie. It is inoculated together with a lactic started culture or, alternatively, sprayed on the product and grows on the surface of the cheese piece [144].

*P.* roqueforti can produce as secondary metabolites components which fall within the category of mycotoxins [145]. Today, starter cultures are available which do not have the ability to produce most of these mycotoxins, and the presence of roquefortine C and isofumigaclavines in the amounts usually found on cheeses does not seem to represent a risk for human health [146]. Alternatively, they are unstable in cheese and are converted to PR-imine [147,148,149].

As an answer to the main concern regarding the use of fungi as ripening agents and the possibility of mycotoxins production, starter cultures do not have the ability to produce most of the above mentioned mycotoxins [146].

#### 2.2.3. Yeasts

In general, yeasts and their metabolic products have been used in different forms of food processing and preservation worldwide, mainly for baking and brewing [19,38]. Nowadays, yeast biotechnology is a part of commercially important sectors, including foods, beverages, pharmaceuticals and industrial enzymes, among others [150].

Of the most isolated strains, *Candida* etchellsii, C. intermedia, C. maltosa, C. versatilis, C. zeylanoides. Candida famata (anamorph of Debaryomyces hansenii), Cyberlindnera jadinii, Cyberlindnera mrakii. Kazachstania unispora, Kazachstania exigua, Pichia kudriavzevii, Kluyveromyces marxianus, Pichia membranefaciensZygotorulaspora florentina [151], Clavispora lusitanae, Cystofilobasidium infirmominiatum, Hanseniaspora uvarum, Kazachstania turicensis, Metschnikowia pulcherrima, Pichia occidentalis, Rhodosporidium sp., Saccharomyces pastorianus, Saccharomycopsis fibuligera, Saturnisporus saitoi, Sporobolomyces roseus, Torulasporadelbrueckii, Trichosporon cutaneum, Trichosporon brassicae, Wickerha-momycesanomalus, Yarrowialipolytica, Zygosaccharomyces bailii, Z. rouxii and Dekkera bruxellensis (anamorph Brettanomyces bruxellensis) [152]. Candida krusei, C. parapsilosis, Candida rugose, P. anomala, Pichia membranifaciens, R. glutinis, S. cerevisiae, Candida boidinii, P. anomala, P. membranifaciens, T. delbrueckii, K. marxianus and D. Hansenii [153,154,155] are the most representative yeasts isolated from Spanish green olives and Greek-style black olives [156,157]. Candida humilis, Kazachstania exigua, Wickerhamomyces anomalus, Candida famata and S. cerevisiae are the most common yeasts strains, which are responsible for the fermentation of sourdough [158].Yeasts’ contribution to the production of fermented beverages cannot be overemphasized. The wide range of these products extends from various ethnic fermented milks to alcoholic drinks, wines and beers. Species like Saccharomycopsis, Pichia, Candida, Torulopsis and Zygosaccharomyces are involved in these fermentations [159]. Kluyveromyces, Saccharomyces and Torula as part of a microorganism’s mixture contribute to the development of sensory characteristics during the fermentation process of products such as kefir [160]. Debaryomyces hansenii and Yarrowia lipolytica are very important for aroma formation in Munster and Parmesan cheeses [161]. Saccharomyces cerevisiae, Hanseniaspora uvarum, Kluyveromyces marxianusa and Pichia fermentans are extremely important for the development of the fine aroma of cocoa beans [151,152].

A brief statement emphasis should be made on the interactions between yeasts and bacteria being convoluted at the fermented food matrix. To strengthen this assumption, the findings of the study by Suharja et al. (2012) are presented [162], who studied the viability of probiotic *Lactobacillus rhamnosus* HN001 in fermented milk with and without *Saccharomyces cerevisiae* var. *bayanus* EC-1118.The authors demonstrated that the yeast supernatant was able to enhance the survival of the tested bacteria in the process and expressed the hope that the findings would help develop novel microbial starter technology for ambient-stable fermented milks with live probiotics.

## 3. Types of Fermented Food

Several categories classifying fermented foods and beverages can be suggested by evaluating (a) the main raw material/s, (b) the timing of the fermentation process, (c) the place of the fermentation process (using the term “territoriality,” as well as certain environment conditions and (d) the intentional application of microbes utilized [17,19,38].

For practical purposes, the classification based on the raw materials and final products was chosen for this review because it shows the wide variety of fermented foods and beverages. In addition, it stresses the diversity of the microbiomes in the food and beverage domain.

### 3.1. Fermented Meats

Fermentation modifies raw meat and transforms it into a different product with new organoleptic characteristics. The key point is the acidification, which enhances protein coagulation (enhancing flavour and softness of the tissues) and drying (lowering the pH also lowers the water activity, a_w_). Meat’s indigenous flora is affected by a series of factors, such as the animal species, breed, nutrition, husbandry, slaughter conditions and post-slaughter preservation. It consists of lactic acid bacteria (mainly *lactobacilli*), coagulase negative staphylococci (CoNS), micrococci and yeasts which can initiate fermentation. Their population size varies significantly from lot to lot, and this makes the control of the process—particularly for artisan products—seem almost impossible [163,164,165]. A typical example is *luza,* a fermented pork meat made in the islands of Aegean archipelago in Greece. The loin is put in a casing (usually made of the large intestine) and is then left suspended to the air for 2 or 3 weeks to dry and to be fermented by its own native microflora. Small amounts of salt and spices are usually added before the casing. The whole process traditionally takes place during winter, during which the strong northern winds and the relatively low temperature optimize the conditions of fermentation. Southern winds with increased air humidity and higher temperatures deflect the fermentation path because of yeasts’ overgrowth and either destroy the product or at least significantly delay the process.

The industrial production cannot rely on the indigenous microflora of meat to produce dry or semidry fermented sausages (the most typical and most popular fermented meat product in the market) because—for obvious reasons—it cannot afford these fluctuations. The process here is quite different from artisan production in many aspects. In the finely grinded meat, salt and spices are added, as well as a small amount of nitrite (to avoid Clostridium vegetation) and a small amount of a fermentable carbohydrate. The latter addition is necessary because meat’s carbohydrates are not enough to feed the fermentation flora. The major technological intervention is the addition of starter cultures of microorganisms (at 10^7^ CFU/g), which rapidly acidify and ferment in a controllable mode. *Pediococcus acidilactici, Pediococcus pentosaceus, Lactobacillus pentosus, L. casei, L. curvatus, L. plantarum* and *L. sakei* are the most common bacteria of these starter cultures. The product is anaerobically cased and left to ferment. The pH value in the commercial form is less than 5.0.

In general, the microbiome of fermented meats (i) provides a long storage period, (ii) ensures safety from pathogens due to its protective activity, (iii) increases tenderness and palatability, (iv) delivers a high nutritional status due to its enzymatic activity and (v) reduces the cooking time [163,164,165].

### 3.2. Fermented Dairy Products

In 2018, the EU yielded 172.2 million tons of milk, 37.7% (64.9 million tons) of which was fermented to cheese and 4.3% (7.4 million tons) was fermented to other products (acidified milk) (ec.europa.eu 24 October 2020) [166]. The figures are impressive by themselves and show, beyond any reasonable doubt, the dynamics of the dairy fermentations market. However, these statistics do not reveal the variety of these products. A few thousand types of cheese are produced across Europe, a variety owing an impressive diversity of microbiomes. From a similar raw material (97% cow’s milk), the different microbiomes lead various pathways of fermentation to produce thousands of different products.

All milk fermentations do not lead to cheese. Kefir, for example, is fermented milk, originally made in Caucasus Mountains from goat’s milk but is nowadays also made from ewe’s milk or even from cow’s milk. The kefir culture typically contains *Lactococcus lactis, Lactobacillus bulgaricus* and *Saccharomyces kefir* and is usually preserved in its dehydrated form as grains (kefir grains). Modern kefir products contain an enriched flora with probiotics such as *Bifidobacterium bifidum, Lactobacillus acidophilus, Lactobacillus helveticus* and others. The end products of the fermentations are lactic acid, ethanol and CO_2_, as well as some other compounds adding to flavour and aroma [20,160].

Greek yoghurt is an extremely popular product in the western markets. It is also called strained yoghurt because whey and other liquids are removed from the traditional yoghurt to produce it. As a result, the Greek yoghurt contains more proteins, less sugars and almost the same amount of fat per 100 gr. The basic culture is homofermentive and contains *Streptococcus thermophillus* and *Lactobacillus bulgaricus*, but in many products under that commercial name, the microbiome is enriched with the addition of probiotic bacteria of the genera *Bifidobacterium, Lactobacillus* and *Lactococcus*. The major product of the fermentation is lactic acid and, in smaller concentrations, other compounds such as diacetyl, acetaldehyde, volatile fatty acids and free aminoacids. These compounds are responsible for its exceptional sensorial characteristics [167].

Starter cultures are a technological intervention widely used in the industrial scale to lead the fermentations to a certain result, but artisan cheeses must rely on the environment. “Tsalafouti” is a Greek cheese made mostly of ewes’ and, in some cases, it may also originate from goats’ milk. Its technology is very simple and serves as an example of the fermentation capacity of natural microflora. The milk is heated until inflation and, after the addition of a small amount of salt, it is left in a cool and shady place for 5–6 days to acidify and to transform into curd by the naturally occurring microbiome of the environment. The cheese is white and creamy, and its flora consists mainly of lactic acid bacteria, namely lactobacilli [46].

The environmental enrichment of the microbiome can be more complicated as in “Kopanisti,” a traditional Greek cheese made in the islands of Aegean Archipelagos. The milk after moderate heating (40–50 °C) and the addition of salt and rennet is left for 3–4 days to form a curd which is taken to a basement or any other cool and shady place. Extra salt is added and mixed thoroughly in the curd with “baking-like “movements,” After 2–3 days, a thin layer of *Penicillium* vegetates all over the surface of the curd. The “baking-like movements” are repeated and the *Penicillium* layer is mixed with the curd. This step is repeated two or three more times and, by then, the cheese is ready. The microbiome of “Kopanisti” consists of an impressive variety of lactic acid bacteria (17 species of lactobacilli were isolated) as well as *Penicillium* spp. Lactic acid bacteria originate from the raw milk, as well as from the environment as, similarly to *Penicillium* spp. This kind of environmental enrichment renders artisan cheeses to “sensitive indicators” of the microbiological ecosystem in which they are produced. It is this enrichment that differentiates artisan cheeses from industrial ones by conferring an original flavour and aroma to them while creating serious safety issues [53].

These four products were select among numerous others as paradigms of the diversity of the fermentations of milk and to underline the role of the environmental enrichment of the microbiome. Dairy products are considered very beneficial to health not only for their nutritional value, but also for their microbiome, which exerts several probiotic functions, thus enhancing the health of the consumer.

### 3.3. Sourdough

It is admirable when two simple ingredients, flour and water, and minimal preparation and process are all that are needed to create such an elegant and “alive” final product, sourdough. This unique simplicity, as the main characteristic of fermentation, is “from art to science.” However, behind the simplicity lies a complex microbial ecosystem, mainly represented by the species of LAB and yeasts [168] a sym-combiota that, through fermentation, confers flavours, aromas, shelf stability and even nutritional features to the resulting products, dough and bread preparation [169]. The sourdough is a famous traveller of the world, with versions of sourdough in Northern Italy, Milan, and San Francisco, as well as selroti and “dhokla” in India; idli in India and Sri Lanka; dosa in India and Sri Lanka; kisra in Sudan; “jalebi” in India, Nepal, and Pakistan; and trahanain in Turkey, Cyprus and Greece [40,170,171,172,173,174,175,176].

Sourdough, as mentioned above, is a mixture of flour and water which is fermented under the influence of lactic acid bacteria (LAB) and mainly heterofermentative strains (when the backslopping method is used) and under the interaction of the produced lactic acid and acetic acid in the mixture, promoting the distinct sour taste of the final product [177].

Dough acidification is an important and even a mandatory step for (a) deactivation of the flour’s a-amylase and (b) activation of the cereals’ phytases, so that the nutrient compounds become bioavailable. Furthermore, along the pathway of sourdough fermentation and afterward at the dough stage, several metabolic processes “come alive,” such as the solubilisation of rye pentosans (reinforced the water binding of the dough), the formation of volatile aromatic compounds and aroma precursors and the production of certain compounds possessed with antimicrobial and antifungal activities, which potentially affect the bread texture, staling and/or shelf-life. [177].

The bacterial and fungal species present in sourdough originate from (a) the microflora of the flour, (b) the bakeries’ environment microbiota, (c) the water used and, according to some researchers, (d) the bakers themselves (their bodily microbiome) [178]. Typically, in the biomass of sourdough, it is mostly the *Lactobacillus* strains producing acid and yeasts who undertake the production of carbon dioxide. Some studies, however, have highlighted a different population dynamic and point to a greater variety of bacteria and yeasts other than *Lactobacillus* and *Saccharomyces* [179,180,181,182]. Other studies have revealed that, at the genus level, *Enterococcus*, *Lactococcus*, *Lactobacillus*, *Leuconostoc*, *Pediococcus*, *Streptococcus* and *Weissella* are associated with sourdough production [177,178]. In the above micro-ecological niche, the genus *Lactobacillus* is dominant. A native strain of *S. cerevisiae* is the principal yeast of most bread fermentations [183]. However, several non-*Saccharomyces* yeasts occur in the fungal consortia in sourdough fermentation, such as *Candida*, *Rhodotorula*, *Pichia*, *Trichosporon*, *Sporobolomyces* and *Yarrowia* [184,185,186]. Prevalent strains show a biodiversity in different geographical areas, including *L. plantarum*, *L. sanfrancisensis*, *L. pontis*, *Lb brevis*, *L. curvatus*, *Lb. sakei*, *L. panis*, *L. alimentarius*, *L. paralimentarius*, *L. pentosus*, *L. helveticus, L. casei, L. amylovorus, L. buchneri, L. farciminis, L.fructivorans*, *L. homohiochii, L. hilgardii, L. johnsonii, L. lactis* subsp. *lactis, L. crispatus*, *L. delbrueckii*, *L. fermentum*, *L. reuteri*, *L. acidophilus*, *L. vaginalis*, *L. gallinarum, L. graminis, Lc. lactis, Leuc. Mesenteroides* subsp. *mesenteroides, Leuc. Mesenteroides* subsp. *dextranicum, Lc. holzapfelii, P. pentosaceus*, *W. cibaria, W. confuse* and *W. viridescens*, with the yeasts *S. cerevisiae, S. exiguus, C. humilis, C. milleri, Issatchenkia orientalis*, *Kazachstania barnettii* and *Saccharomyces bayanus/Kazachstania* sp. [177,178,187].

Although some sourdough contains a few dominant taxa, strain differences and their dynamics during processing can be extremely complex. The actual pathway of the fermentation depends on several endogenous and exogenous factors, such as the type of cereal, leavening and maintenance temperature.

A research about the bacterial ecology during rye and wheat sourdough preparation used 16S rRNA gene pyrosequencing. Several flours were initially contaminated by metabolically active genera (*Acinetobacter*, *Pantoea*, *Pseudomonas*, *Comamonas*, *Enterobacter*, *Erwinia* and *Sphingomonas*) belonging to the phylum Proteobacteria or Bacteroidetes (genus *Chryseobacterium*). One day later, the initial population was almost completely inhibited except for the Enterobacteriaceae. Although members of the phylum Firmicutes were present at very low or moderate populations’ size in the flours, they became dominant soon after the first day. The lactic acid bacteria were almost the exclusive representatives of the Firmicutes and (following *Saccharomyces cerevisiae*) became dominant throughout the sourdough proliferation [180].

Another study researched the major problem of the bakery industry, the fungal contamination of bread, a spoilage state that poses safety hazards due to its negative impact on the sensorial qualities of the product and on the potential occurrence of mycotoxins. In that research, different moulds belonging to the genera *Aspergillus* spp., *Penicillium* spp. and *Fusarium* were used to artificially contaminate bread produced with two experimental modes: (i) Inoculation of the dough with a commercial *Saccharomyces cerevisiae* strain (control) and (ii) co-inoculation of the dough with the commercial *S. cerevisiae* strain and with *L. plantarum* UFG 121. The results indicate the potential use of *L. plantarum* UFG 121 in the biomass of the dough as a biocontrol agent in bread production and suggest a species- or strain-depending sensitivity of the moulds to the same microbial-based control strategy [188].

Finally, there is research which deserves special mention and which has negotiated the naturally occurring microbial communities entering in the sourdough mass through the environment and, particularly, from the bakers’ hands. Bakers from two continents used a standardized recipe and the same ingredients to make starters that were then baked into breads. Fungi and bacteria associated with the starters, bakers’ hands and ingredients were characterized using 16S and internal transcribed spacer (ITS) rRNA gene amplicon sequencing. Starter communities were much less uniform than expected, and this variation manifested in the flavour of the bread. The microbiota of starters were more like those found in flour but shared some species with the bakers’ skin. On the contrary, humans are likely to contribute the presence of the microorganisms to the starters, and it also appears that some of them originate from the bakers’ bodily microbiome. This bidirectional exchange of microorganisms between starters and bakers highlights the importance of microbial diversity on bodies and in our environments as it is related to food. Sourdough starters are complex communities of yeast and bacteria which confer characteristic flavour and texture to sourdough bread. The results indicate that bakers may be a source for yeast and bacteria in their breads and/or that those bakers’ occupations are reflected on their skin microbiome [178].

Kara Ali [189] studied the production of the biomass of *S. cerevisiae* on an optimized medium using date extract as the only carbon source in order to obtain a good yield of the biomass. The biomass production was carried out according to the central composite experimental design (CCD) as a response surface methodology.

### 3.4. Fermented Beverages

Fermented beverages include a wide variety of very popular and very famous products. Depending on the substrate and the microorganisms, involved fermentations can take different paths, resulting in different end products. One way to classify them is in two major categories: Alcoholic (more than 0.5% *v*/*v* alcohol) and non-alcoholic beverages (less than 0.5% *v*/*v* alcohol). A special category of alcoholic beverages are the ones with low alcoholic content (no more than 1.2% *v*/*v* alcohol).

However, another perhaps more productive way for classification is on the basis of the sources (substrates) from which they originate. There are five main categories of fermented beverages depending on the raw material: (i) From grains (barley, corn, rye, millet etc), resulting in products such as Irish whiskey, beer, bourbon whiskey, boza, kvass and vodka; (ii) from fruit juice (apples, bananas, cherries, grapes, ginger, etc.), producing cider, wines, cherry wine, ginger beer and ginger ale, among other products; (iii) from vegetables (potatoes, sugarcanes, sauerkrauts, etc.), producing vodka (in Poland), rum, sauerkraut juice, etc.; (iv) from milk, producing many beverages such as ayran, koumis, kefir and buttermilk; and (y) other raw materials, such as tea leaves, honey, sugar and palm sap, producing various beverages (kombucha, mead, etc.) [190].

Some of these ferments, e.g., wines, are usually consumed for recreational purposes, although, if consumed in reasonable quantities and frequency, they may have certain beneficiary effects to health. Other ferments, however, such asayran, kefir or boza, have exceptional nutritional qualities [191].

Different fermented vegetables have been described by Varzakas et al. [192], for example, soybean tempeh and other soybean paste products, sauerkraut, fermented olives, fermented cucumber and kimchi. In addition, salting procedures were explained along with the role of lactic acid bacteria in fermented vegetables. Regarding sauerkraut, Zabat et al. [193] utilized 16S rRNA amplicon sequencing to profile the microbial community of naturally fermented sauerkraut throughout the fermentation process and analysed the bacterial communities of the starting ingredients and the production environment. They showed that the sauerkraut microbiome is rapidly established after fermentation begins and that the community is stable throughout fermentation and packaging for commercial sale.

Moreover, Bell et al. [194,195] highlighted the role of fermented foods and beverages on gut microbiota and debated the need for transdisciplinary fields of One Health if communication is to be enhanced. They addressed nutritional and health attributes and reported that they are not included globally in world food guidelines. They also mentioned some traditional African fermented products.

## 4. Importance of Fermented Foods in Health and Oral Health

Probiotics and prebiotics are now widely used in both medical (such as to lower cancer risk and protect gastrointestinal tract health and urinary and vaginal tract health) and dental specialties (to reducecaries development, lower the risk of dental erosion, achieve periodontal health, reduce oral malodour, etc.). Thus, a thorough understanding of their risks and benefits is essential.

From general gut health to immune support, skin health, cholesterol control, lactose intolerance and maybe even sensorimotor behaviour, the research is continually building. Over the past decade, there has been extensive work in animal models on how probiotics and prebiotics modulate host metabolism. Studies with animal models have shown that the gut microbiota can regulate inflammation, adiposity, satiety, energy expenditure and glucose metabolism. As more knowledge on the mechanisms from in vivo experiments is unravelling, there is a growing need to translate the results to discover the potential human health benefits. However, there are very few good, double-blind, placebo-controlled clinical trials that have proven the effects of pro- and prebiotics on modulating human metabolism [196]. At present, high-quality human trials have demonstrated the potential for gut microbiota in manipulating and preventing or treating diseases such as hypercholesterolemia and obesity [197]. It has been further investigated that *B.breve*, *B*. *bifidum*, *B.pseudolongum* and *Lactobacillus* convert linoleic acid (LA) into conjugated linoleic acid, which suppresses multistage carcinogenesis at different sites [198]. *L.helveticus* and *B.longum* have also been reported to produce and respond to mammalian serotonin and affect behaviour modulation [199,200]. The ability of these bacteria to produce and respond to neurochemicals substantiates the potential of probiotics to influence psychological health and general behaviour [200]. It is therefore probable that modulating the gut microbiota with such biotherapeutics may target stress-related CNS disorders, including stress-induced cognitive deficits [201]. However, the elucidation of mechanisms and substantiation of animal studies in humans remain essential research goals and need further consideration.

Furthermore, great progress has been achieved in our understanding of the prevalence and diversity of the microbial communities within the oral cavity itself, as well as their result in the healthy physiology of the mouth. Studies on the microbiota indicate that other microorganisms, specifically fungi, are also an important part of the oral microbiome which in part can be strongly associated with serious oral colonization diseases such as tooth decay (dental caries), gum disease (periodontitis) and mucous membrane diseases (e.g., candidiasis). To prevent these diseases, the consumption of fermented foods has been suggested [202] since they contain probiotic bacteria as well as prebiotic fibers that feed the human mouth flora and help the positive interactions between the approximately 600 prevalent bacterial species of the human mouth [203].

The mechanisms of action explaining beneficial probiotic effects include modulation of host immune response leading to strengthening of the resistance to pathogenic challenge and alteration of the composition and metabolic activity of host micro-flora at the specific location [204]. What the probiotics offer is: (1) Adhesion and colonization (at least transitory) in the human body. Adhesion may increase the retention time of a probiotic and place bacteria and host surfaces (body fluids and epithelial cells) in close contact, thus facilitating further probiotic activity, (2) Enhancement of the non-specific and specific immune response of the host, (3) Production of antimicrobial substances and competition with pathogens for binding sites, (4) Survival and resistance to human defence mechanisms during the oro-gastro-intestinal transit and (5) Safety to the macro-organism [205]

**Figure 2:**
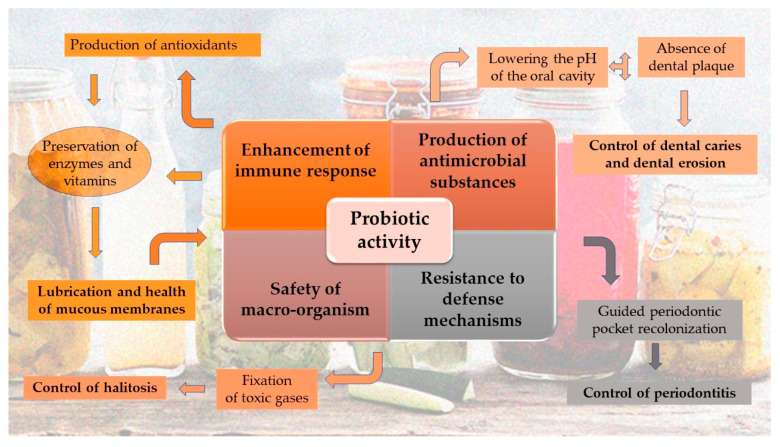
Mechanisms and advantages of action of fermented foods in the oral cavity.

Under this scope, the treatment of periodontal diseases and dental caries, has moved towards an antibiotic/anti-microbial model of disease management and in this sense, probiotics might be a promising area of research in the treatment of these modalities. To explain more the connection, probiotics decrease the pH of the oral cavity due to the chemistry of *Lactobacillus* bacteria which can convert carbohydrates (sugars) into lactic acid, so that plaque bacteria cannot form dental plaque and calculus, thus diminishing the risk of these diseases but also the risk of erosion of the hard tooth tissues [204]. The property of probiotics of neutralizing acidic condition and decreasing the pH of the oral cavity, helps in the management of dental caries and is the reason why they are nowadays incorporated in the dairy products. The lactic acid also naturally stunts the growth of harmful bacteria and increases or preserves the enzymes and vitamins for better digestion [206]. The absorption of vitamins is very important for the lubrication, the good preservation of the mucous membranes and the sanity of gum tissues [202,205,206]. They also produce antioxidants which prevent plaque formation by neutralizing the free electrons that are needed for the mineral formation and concluding in the sanity of the oral membranes. Probiotics can also breakdown putrescence odors by fixating on the toxic gases (volatile sulfur compounds) and changing them to gases needed for metabolism [207] thus treating halitosis. Further, guided pocket re-colonization approach with the use of probiotics may provide a valuable addition or alternative to the armamentarium of treatment options for periodontitis [208]. Finally, the efficacy of probiotics on dental caries has been studied worldwide utilizing different strains species of *Lactobacillus* like *L. rhamnosus* GG and *L. casei* which inhibit the growth of oral streptococci. As it is well-known, *S.mutans* is the most common organism that leads to development of caries [209]. For the above reasons dairy products are suggested for consumption immediately after lunch or dinner for the prevention of dental caries and erosion in a daily base [206] (Figure 2: Mechanisms and advantages of action of fermented foods in the oral cavity).

Based on the above information, the concept of probiotics has opened new horizons on the relationship between diet, health and oral health. Exploring and reporting the type, quantity and way of consumption of fermented foods in the daily routine of all age groups will be in the scopes of future in vivo research in humans.

## 5. Safety Issues

One could argue that microbiomes can protect their food ecosystems themselves. Indeed, it seems that microbiomes can deploy certain defence lines and hence rapidly transform their environment in such a way that it finally becomes hostile to most other microorganisms. The key words are “finally” and “most.” It is true that fermentation itself serves as the first line of defence by rapidly decreasing the pH values (the involved microorganisms are also called “fast acidifiers”) through the production of organic acids (e.g., lactic acid) and also by eliminating the carbohydrates of the environment, thus depriving the competitive bacteria of nutrients. However, this process—no matter how fast it proceeds—takes some time, and this provides a window of opportunity to various spoilage intruders or pathogens. This is particularly true for fresh cheeses of artisan origin. Various *Brucella* strains manage to survive in such products, causing serious foodborne illnesses, although they are eliminated in cheeses with a ripening period longer than 2 months [210]. Normally, by the end of the ripening process, most pathogens and spoilage bacteria will be destroyed or permanently inactivated due to the low pH, deprivation of nutrients and antibacterial activity of certain substances produced by the fermentation flora. Staphylococcal species, on the other hand, survive throughout the fermentation process, though a healthy microbiome can control effectively their populations [211].

### 5.1. Bacteriocins

Besides organic acids, certain species of microbiome communities, and particularly the lactic acid bacteria (LAB), excrete molecules with antimicrobial action such as hydrogen peroxide, acetaldehyde and bacteriocins. These molecules—products of the bacterial cell metabolism—have been proven effective against pathogens and spoilage bacteria both in vivo and in vitro. The production of bacteriocins is among the criteria for a strain to be characterized as probiotic [212].

Bacteriocins are proteinaceous molecules (peptides) able of destroying other bacteria. They are synthesized in the ribosomes and are excreted extracellularly, killing mainly—but not exclusively—Gram-positive bacteria [213,214,215]. They have a narrow or broad spectrum of activity depending on the number of sensitive species. All bacteriocins do not possess the same antibacterial capacity, and all bacteriocin-producing bacteria do not produce the same amount of bacteriocins of the same potency. Their activity depends on their exact chemical structure as well as the susceptibility of the target cell [216]. These substances attract increasing attention in the food industry because they are a natural means of preservation of products against pathogens and spoilage bacteria. The utilization of bacteriocins can potentially lead to milder treatments or minimal processing. Bacteriocins are not toxic and they do not alter the sensorial characteristics of the products. These advantages meet the expectations of the consumers for “natural” foods and less chemicals. They also satisfy the necessity of the food industry to satisfy these demands. Thus, bacteriocins fit perfectly into the concept of “biopreservation” [217].

Bacteriocins can either be added directly as chemical substances to the products or can be added indirectly through LAB cultures, although it seems that the latter case is not an optimal strategy because the additional LAB could ferment the carbohydrates of the product, thus altering its properties. Nisin, pediosin, enterocins AS-48 and 416K1, bificin C6165 and bovicin are bacteriocins which have been studied for their antimicrobial action. So far, only pediosin and nisin have received official approvals as food additives, yet the research in that scientific field is ongoing and the future of bacteriocins in the food industry is promising [215].

### 5.2. Antibiotic Resistance

Resistance to antibiotics (AR) is globally recognized as an emerging serious threat to human health and as a food safety problem (FAO/WHO 2018, WHO 2020) [218]. The term refers to bacterial strains (pathogens or not) that are resistant to one or more antibiotics (multidrug resistant, MDR). It is estimated that AR causes at least 700,000 deaths worldwide [219]. In the EU, there is an officially expressed concern for the observed increased resistance to first-line antibiotics such as carbapenems and quinolones [220]. In the 2019 CDC report on AR, it was estimated that, in the US, 2.8 million people are infected by resistant bacteria while 35,000 die as a result of these infections (CDC 2020) [221].

Foods, and particularly fermented foods, are ecosystems favouring bacterial growth. The ecology of food natural flora is very complicated and can originate from various niches. Fermentations’ microbiomes are a heterogenous group in the sense that the bacterial species colonizing such foods occur from diverse origins, such as plants and their soil, the animals’ udder or gut, the environment in which the animals live (terrestrial or aquatic), the facilities of the food processing (dairies, abattoirs, etc.) and the retail and distribution networks. In this way, various ecological niches are represented in the biota of fermented milks, meats and vegetables. In these niches, bacteria and AR genes co-circulate. The so-called mobile genetic elements (MGE), R plasmids, integrons and transposons, as well as bacteriophages, carry the genetic determinants of AR and disperse them among bacterial communities [222]. Hence, AR is acquired from pathogenic and non pathogenic bacteria. In the former case, the threat to the consumer’s health is straightforward and represents a medical emergency. However, it is the latter case that poses a more subtle and potentially more dangerous health hazard. Non pathogens and commensal bacteria such as these that are found in the microbiomes can easily acquire resistance genes from other bacteria through MGE and can confer them to the consumer’s intestinal microbiome. Thus, even a non-resistant pathogen (foodborne or not) can receive resistance genes in the human digestive tract [222].

LAB bacterial species dominate the flora of fermented foods and most of them, such as *Lactobacillus* spp., have been shown to be resistant to a variety of antimicrobial substances representing all categories of antibiotics, thus posing a potential danger [45]. Regulation through legislation for the prudent use of antibiotics in agriculture, husbandry and aquacultures is necessary to tackle this issue. Regulation should also be imposed in the food industry concerning the resistance to antibiotics.

Antibiotics are used as therapeutic means against infectious disease in humans, animals and aquaculture. However, the haphazard and extensive use of antibiotics may lead toantibiotic-resistant bacteria [223]. The development of resistance to antibiotics can be established by several mechanisms, that is, by enzymatic degradation, by modifying the target of the antibiotic, by affecting the permeability of the bacterial cell wall and by creating alternative pathways to elude the activity of the antibiotic [224]. Some species exhibit inherited or innate resistance to certain antibiotics, but such resistance cannot be transferred to other bacteria. Acquired antibiotic resistance is observed when bacteria acquire genes encoding a resistance mechanism either from other bacteria or by mutations [225].

Fermented foods and beverages are produced worldwide, and they are valued for their sensorial and nutritional characteristics. Numerous studies have shown that the beneficial bacteria of these ferments are also carriers of resistance to antibiotics [226]. The food chain is an immense route and the fermented foods a subroute through which the determinants of resistance to antibiotics are conferred to pathogens and to commensal bacteria [227].

LAB particularly is abundant in fermented foods and may act as reservoirs of antibiotic resistance genes, which, in turn, can be horizontally transmitted to pathogens and to the consumers’ microbiome via the food chain [228,229].

Antimicrobial resistance genes can be transferred into microbial communities by mobile genetic elements (MGE) such as plasmids, transposons, integrons, cassettes or bacteriophages [230]. The European Scientific Committee on Animal Nutrition and the European Food Safety Authority recommend that LAB strains consumed daily should lack acquired or transferable antimicrobial resistance genes in order to be considered safe for human and animal consumption [231].

## 6. Conclusions

Fermentations dominate the history and present of nutrition in the sense that, in one form or another, most humans eat (or drink) fermented foods daily. There is a vast variety of fermented foods and drinks throughout the world, some of which were indicatively discussed. The multitude of microorganisms involved in fermentations—associated with complicated metabolic pathways—is more than impressive. Apart from their nutritional effects, fermented foods enhance and protect the health of the consumer in more than one way. Most of these microorganisms extend their action into digestive tract of the consumer, conferring serious benefits to his/her health. This probiotic effect has been extensively verified in vivo and in vitro by numerous reports. Regular consumption of fermented foods reduces the risk of certain types of cancer, protects the health of the gastrointestinal tract and the urogenital tract, alleviates symptoms of diseases such as Crohn’s disease and irritable bowel disease and enhances the immune system. There are strong indications of a positive impact to certain psychological and behavioural disorders. There is also a certain beneficial effect of their daily use to the oral health. By lowering the pH value in the oral cavity, they prevent dental caries and the erosion of teeth while contributing to the treatment of periodontitis and halitosis. The lowering of the pH value of the fermented products is a defence mechanism of the microbiome, since most antagonistic bacteria cannot survive at low pH values. Bacteriocins are an effective means of overcoming other bacteria. These substances are a very promising tool in the food industry because they are a natural way of preservation, fully compatible with consumers’ expectations for a mild treatment of food. Yet, the increasing incidence of resistant strains in the microbiomes is a trend that should be addressed by the scientific community.

## Figures and Tables

**Table 1:** Most common fermentations and the implicated microorganisms.

Type of Fermentation	Microorganisms Involved	Food/Environment	Major End Products
Alcohol fermentation	Yeasts	Wine, beer, sourdough	*2 glucose → 2 glycerol + acetic acid + ethanol*
Lactic acid (homofermentation)	Lactic acid bacteria (*Lactobacillus* spp. etc.)	Dairy products, fermented meats and fermented vegetables etc.	*Glucose → 2 lactic acid + 1ATP (Embden-Meyerhof-Parnas)*
Lactic acid (heterofermentation)	Lactic acid bacteria (*Lactobacillus* spp. etc.)		*5 and 6 carbon sugars → lactic acid + acetic acid+ ethanol +1ATP*
Butyric acid	*Clostridium* spp., *Butyrivibrio* spp., *Bacillus* spp. *and other anaerobes*	Marsh sediments, sewage systems	*4 glucose → 2 acetate + 3 butyrate → butyric acid, butanol, acetoine, isopropanol, acetate, ethanol, 2.3-butadienol*
Mixed acid	Enterobacteriaceae *(Escherichia* spp., *Enterobacter* spp., *Salmonella* spp., *Klebsiella* spp., *Shigella* spp. *etc.)*	Human and animal digestive tract, fresh water	*Glucose → acetic acid, formic acid, lactic acid, succinic acid, ethanol*
Propionic acid	*Propionebacterium* spp., *Veilonella* spp., *Bacteroides* spp., *some Clostridia* spp.	Dairy products	*Glucose, glycerol, lactate → propionate, acetate,*
Acetic acid	*Acetobacter* spp., *Gluconobacter* spp., *Bacillus subtilis*	Acetic acid industry	*Oxidation of sugars, sugar alcohols, ethanol →acetic acid*

**Table 2:** Bacterial diversity implicated in fermentations pathways.

*Phylum Proteobacteriaceae*			
Family	Genus	Species	Products/Food Usage
Acetobacterraceae	***Acetobacter***	*A.aceti* subsp*aceti*, *A. oryzae*, *A. pasteurianus* (subsp*pasteurianus*), *A. polyxygenes*, *A. xylinum*, *A. malorum*, *A. pomorum*, *A. syzgii*, *A. fabarum*, *A. lovaniesis*, *A. orientalis*, *A. tropicalis*	Mainly in vinegar but also isolated from some other fermentation products of cocoa, coffee and tea [87,91,92]
	***Gluconacetobacter***	*G. azotocaptans*, *G. diazotrophicus*, *G. entanii*, *G. europaeus*, *G. hansenii*, *G. johannae*, *G. oboediens*, *G. oxydans*, *G. xylinus*	
**Enterobacteriaceae**	***Halomonas***	*H. elongate*	Meat fermentation [19,87,93]
	***Hafnia***	*H. alvei*	Dairy fermentation [19,87]
Erwiniaceae	***Tatumella***	*T. citrea*, *T. ptyseos*	Cocoa bean fermentation [94]
**Sphingomonadaceae**	***Zymomonas***	*Z. mobilis* subsp*mobilis*	Fermentation process of alcoholic beverages in many tropical areas of America, Africa and Asia [87,95,96]
***Phylum Actinobacteriaceae***			
**Family**	**Genus**	**Species**	
**Dermabacteraceae**	***Brachybacterium***	*B. alimentarium*, *B. tyrofermentans*	Major part of the surface microbiota of certain types of cheese (Gruyere and Beaufort cheese) [97]
**Microbacteriaceae**	***Microbacterium***	*M. gubbeenense*	Important to the traditional red smear surface culture of surface-ripened cheeses [98]
**Brevibacteriaceae**	***Brevibacterium***	*B. casei*, *B. linens*, *B. aurantiacum*	Smear-ripened cheeses [99]
**Corynebacteriaceae**	***Corynebacterium***	*C. ammoniagenes*, *C. casei*, *C. flavescens*, *C. variabile*, *C. manihot*	Dairy products *C. manihot* dominates at the initial stage of cassava (a traditional staple food) fermentation [98]
**Micrococcaceae**	***Micrococcus***	*M. luteus*, *M. lylae*	Cheese ripening the former and meat fermentation the latter [87,100]
	***Kocuria***	*K. rhizophila*, *K. varians*	Traditional dairy and meat fermentation the former and as bioactive agent controlling the growth of pathogens in chilled dairy products the latter [87,101]
	***Arthrobacter***	A.arilatensis, A. bergerei, A.globiformis, A. ricotianae	Cheese production [38,87]
**Propionibacteriaceae**	***Propionibacterium***	*P. acidipropionici*, *P. freudenreichii* subsp *freudenreichii*, *P. freudenreichii* subsp *shemani*, *P. freudenreichii* subsp *globosum*, *P. freudenreichii* subsp *germanii*, *P. thoeni*, *P. jensenii*	Dairy products and as secondary microflora in cultured buttermilk [70,87]
**Streptomycetaceae**	**Streptomyces**	S. griseussubspgriseus	Fermented meat products [102]
**Bifidobacteriaceae**	***Bifidobacterium***	*B. bifidum*, *B. adolescentis*, *B. animalis* subsp *animalis*, *B. animalis* subsp *lactis*, *B. longum* subsp *infantis*, *B. theromophilum*, *B. pseudologum* subsp *pseudologum*, *B. breve*	Dairy products, *B. breve* is also used in the production soy products [38,87,103]
***Phylum Firmicutes***			
**Bacillaceae**	***Bacillus***	*B. coagulans*, *B. subtilis*, *B. subtilis var natto*, *B. amyloliquefaciens*, *B. licheniformism*, *B. circulans*, *B. firmus*, *B. megaterium*, *B. pumilus*, *B. thermoamylovorans*, *B. thuringiensis*, *B. macrerans*	Fermentation of cocoa, soy, fish products, “Pidan” (preserved alkali-treated fresh duck eggs, China) and industrial producers of γ- PGA in fermented soybean foods, respectively [37,38,104]
**Sporolactobacillaceae (Staphylococcaceae)**	***Gemella***		Legume undergoing alkaline fermentation [82]
**Staphylococcaceae**	***Jeotgalicoccus***		Legume undergoing alkaline fermentation [82]
**Carnobacteriaceae**	***Carnobacterium***	*C. divergens*, *C. maltaromanticum*, *C. piscicola*,	fish fermentation and in lesser extent in dairy products [19,38,87]
**Enterococcaceae**	***Enterococcus***	*E. durans*, *E. faecalis*, *E. faecium*, *E. cloacae*,	Fermentations of milk, meat, vegetables and cereals [105]
	***Tetragenococcus***	*T. koreensis*, *T. halopilus*	Vegetable fermentation [19,38], soybean paste [106]
	***Vagococcus***		Traditional fermented soybean food in India [107]
**Leuconostocaceae**	***Weissela***	*W. confusa*, *W. beninensis*, *W. ciburia*, *W. fabaria*, *W. ghanensis*, *W. hellenica*, *W. koreensis*, *W. paramesenteroides*, *W. viridencens*, *W. thailandensis*	Fermented vegetables, meat, fish and cocoa, with some of them showing remarkable antimicrobial activity against Gram-positive and Gram-negative pathogens [108]
	***Leuconostoc***	*L. lactis*, *L. mesenteroides* subsp. *cremoris*, *L. mesenteroides* subsp. *dextranicum*, *L. mesenteroides* subsp. *mesenteroides*, *L. pseudomesenteroides*, *L. citreum*, *L. fallax*, *L. holzapfelii*, *L. inhae*, *L. kimchi*, *L. carnosumm*, *L. gasicomitatum*, *L. gelidum*, *L. palmae*	All fields of the fermented food industry [46]
	***Oenococcus***	*Oenococcusoeni* (which was known as *Leuconostoc oeni* until 1995)	Fermented grapes and apple juices, considered as the most frequently detected bacterium during malolactic fermentation [109]
**Staphylococcaceae**	***Staphylococcus*** (Coagulase negative Staphylococci, CoNS)	*S. carnosus* subsp. *utilis*, *S. carnosus* subsp. *carnosus*, *S. cohnii*, *S. condimenti*, *S. equorum* subsp. *equorum*, *S. linens*, *S. fleuretiii*, *S. piscifermentans*, *S. saprophyticus*, *S. sciuri* subsp. *sciuri*, *S. succinus* subsp. *succinus*, *S. epidermidis*, *S. succinus* subsp. *casei*, *S. kloosii*, *S. gallinarum*, *S. vitulinus*, *S. warneri*, *S. xylosus*	Part of the natural microbiota of fermented meat products as well as of hard and of soft cheeses and of fermented vegetables [110,111]
**Streptococacceae**	***Lactococcus***	*L. lactis* subsp*lactis. Lactis* subsp. *cremoris*, *L. gallolyticus* subsp. *macedonicus*, *L. raffinolactis*	Important industrial dairy starter, mainly in the yoghurt production and elsewhere [87,112]
	***Streptococcus***	*S. salivarius* subsp. *salivarius*, *S. salivarius* subsp. *thermophilus*, *S. gallolyticus* subsp. *macedonicus*	Milk fermentation, and in adjacent cultures for cheeses production [113]
**Lactobacillaceae**	***Lactobacillus***	*L. helveticus*, *L. casei*, *L. paracasei*, *L. plantarum/paraplantarum*, *L. pentosus*, *L. salivarius*, *L. rhamnosus*, *L. curvatus*, *L. brevis*, *L. sakei*, *L. acidophilus*, *L. acidipiscis*, *L. reuteri*, *L. johnsonii*, *L. kefiri*, *L. parakefiri*, *L. kefiranofaciens*, *L. nodensis*, *L. crispatus*, *L. fermentum*, *L. delbrueckii*, *L. buchneri*, *L. coryniformis* subsp. *coryniformis*, *L. gasseri*	Fermented dairy products [19,38,87]
		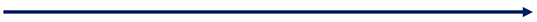	
		*L. acetotolerans*, *L. acidifarinae*, *L. alimentarius*, *L. amolylovorus*, *L. buchneri*, *L. cacaonum*, *L. collinoides*, *L. crustorum*, *L. curvatus*, *L. farciminis*, *L. diolvorans*, *frumenti*, *L. kimchii*, *L. kisonensis*, *L. mali*, *L. mucosae*, *L. nagelii*, *L. portis*, *L. rapi*, *L. sakei* subsp. *carnosus*, *L. vaccinostercus*, *L. tuceti*, *L. surkii*, *L. spicheri*, *L. similis*	Fermented vegetables—fruits, meat—fish and cocoa, sourdough, cereals, beverages [19,38,87].
	***Pediococcus***	*P. acidilactici*, *P. damnosus*, *P. parvulus*, *P. pentosaceus* subsp. *pentosaceus*, *P. pentosaceus* subsp. *intermedius*, *P. clausenii*	Isolated from a variety of foods such as sausages, pickles, fermented cucumber, cheeses, wines and beverages [114,115]

## References

[1] (1879). Modern History Sourcebook: Louis Pasteur (1822–1895): From the Physiological Theory of Fermentation by Louis Pasteur. https://sourcebooks.fordham.edu/mod/1879pasteur-ferment.asp.

[2] Barnett J. (2003). Beginnings of microbiology and biochemistry: The contribution of yeast research. Microbiology.

[3] Albert G., Moat J.W., Foster M.P.S. (2002). Microbial Physiology.

[4] Thomas J., Montville K.R.M. (2008). Food Microbiology. An Introduction.

[5] Toussaint-Samat M. (2009). A History of Food.

[6] Angold R., Beech G., Taggart J. (1989). Food Biotechnology: Cambridge Studies in Biotechnology 7.

[7] Caballero B., Trugo L.C., Finglas P.M. (2003). Encyclopaedia of Food Sciences and Nutrition.

[8] Tokuhiro K., Ishida N., Nagamori E., Saitoh S., Onishi T., Kondo A., Takahashi H. (2009). Double mutation of the PDC1 and *ADH1* genes improves lactate production in the yeast Saccharomyces cerevisiae expressing the bovine lactate dehydrogenase gene. Appl. Microbiol. Biotechnol..

[9] Frutiger M. (2017). The History of Cheese in Switzerland. https://www.swissclubnsw.com/single-post/2017/04/26/The-History-of-Cheese-in-Switzerland.

[10] Homer, ’The Odyssey’ Bk V:192-261. https://www.poetryintranslation.com/PITBR/Greek/Odhome.php.

[11] Prajapati J.B., Nair B.M., Farnworth E.R. (2003). The history of fermented foods. Fermented Functional Foods.

[12] McGovern P.E. (2007). Ancient Wine: The Search for the Origins of Viniculture.

[13] McGovern P.E. (2010). Uncorking the Past: The Quest for Wine, Beer, and Other Alcoholic Beverages.

[14] McGovern P.E., Mirzoian A., Hall G.R. (2009). Ancient Egyptian herbal wines. Proc. Natl. Acad. Sci. USA.

[15] Hukins W.R. (2006). Microbiology and Technology of Fermented Foods.

[16] Hornsey S.I. (2003). A History of Beer and Brewing.

[17] Owusu-Kwarteng J., Akabanda F., Agyei D., Jespersen L. (2020). Microbial safety of milk production and fermented dairy products in Africa. Microorganisms.

[18] Surono S.I., Tamang J.P. (2016). Ethnic fermented foods and beverages of Indonesia. Ethnic Fermented Foods and Alcoholic Beverages of Asia.

[19] Tamang P.J., Thapa N., Tamang B., Rai K.A., Chettri R., Tamang J.P. (2015). Microorganisms in fermented foods and beverages. Health Benefits of Fermented Foods.

[20] Dimidi E., Cox R.S., Rossi M., Whelan K. (2019). Fermented foods: Definitions and characteristics, impact on the gut microbiota and effects on gastrointestinal health and disease. Nutrients.

[21] Chaves-López C., Rossi C., Maggio F., Paparella A., Serio A. (2020). Changes occurring in spontaneous maize fermentation: An overview. Fermentation.

[22] Rezac S., ReenKok C., Heermann M., Hutkins R.W. (2018). Fermented foods as a dietary source of live organisms. Front. Microbiol..

[23] Sivamaruthi S.B., Kesikaand P., Chaiyasut C. (2019). Toxins in fermented foods: Prevalence and preventions—A mini review. Toxins.

[24] Andoh A., Yoshida T., Yagi Y., Bamba S., Hata K., Tsujikawa T., Kitoh K., Sasaki M., Fujiyama Y. (2006). Increased aggregation response of platelets in patients with inflammatory bowel disease. J. Gastroenterol..

[25] Limdi K.J., O’Neill C., McLaughlin J. (2006). Do probiotics have a therapeutic role in gastroenterology?. World J. Gastroenterol..

[26] Young P., Cash D.B. (2006). Probiotic use in irritable bowel syndrome. Curr. Gastroenterol. Rep..

[27] Rolfe V.E., Fortun P.J., Hawkey C.J., Bath-Hextall F. (2006). Probiotics for maintenance of remission in Crohn’s disease. Cochrane Database Syst. Rev..

[28] Boyle R.J., Tang M.L. (2006). The role of probiotics in the management of allergic disease. Clin. Exp. Allergy.

[29] Falagas E.M., Betsi I.G., Tokas T., Athanasiou S. (2006). Probiotics for prevention of recurrent urinary tract infections in women. Drugs.

[30] Regal R., Pham C., Bostwick T. (2006). Urinary tract infections in extended care facilities: Preventive management strategies. Consult. Pharm..

[31] Sudha M.R., Chauhan P., Dixit K., Babu S., Jamil K. (2009). Probiotics as complementary therapy for hypercholesterolemia. Biol. Med..

[32] Zmora N., Zilberman-Schapira G., Suez J., Mor U., Dori-Bachash M., Bashiardes S., Kotler E., Zur M., Regev-Lehavi D., Brik R.B.Z. (2018). Personalized gut mucosal colonization resistance to empiric probiotics is associated with unique host and microbiome features. Cell.

[33] Lavefve L., Marasini D., Carbonero F. (2019). Chapter three—Microbial ecology of fermented vegetables and non-alcoholic drinks and current knowledge on their impact on human health. Adv. Food Nutr. Res..

[34] McNeilL B.M., Harvey N., Rowan N.J., Giavasis I. (2013). Fermentation Monitoring and Control of Microbial Cultures for Food Ingredient Manufacture.

[35] Ravyts F., De Vuyst L., Leroy F. (2012). Bacterial diversity and functionalities in food fermentations. Eng. Life Sci..

[36] Xiang H., Sun-Waterhouse D., Waterhouse I.N.G., Cui C., Ruan Z. (2019). Fermentation-enabled wellness foods: A fresh perspective. Food Sci. Hum. Wellness.

[37] Tamang J.P., Shin D.H., Jung S.J., Chae S.W. (2016). Functional properties of microorganisms in fermented foods. Front. Microbiol..

[38] Tamang J.P., Holzapfel W.H., Watanabe K. (2016). Diversity of microorganisms in global fermented foods and beverages. Front. Microbiol..

[39] Tamang J.P. (2010). Himalayan Fermented Foods: Microbiology, Nutrition, and Ethnic Values.

[40] Tamang J.P., Cotter D.P., Endo A., Han S.N., Kort R., Liu Q.S., Mayo B., Westerik N., Hutkins R. (2020). Fermented foods in a global age: East meets West. Compr. Rev. Food Sci. Food Saf..

[41] Ray C.R., Joshi V.K. (2014). Fermented foods: Past, present and future. Microorg. Ferment. Tradit. Foods.

[42] Tamang B., Tamang J.P. (2008). In situ fermentation dynamics during production of gundrukandkhalpi, ethnic fermented vegetables products of the Himalayas. Indian J. Microbiol..

[43] Lee S.H., Jung J.Y., Jeon C.O. (2015). Source tracking and succession of kimchi lactic acid bacteria during fermentation. J. Food Sci..

[44] Lee S.H., Whon T.W., Roh S.W., Jeon C.O. (2020). Unraveling microbial fermentation features in kimchi: From classical to meta-omics approaches. Appl. Microbiol. Biotechnol..

[45] Rozos G., Voidarou C., Stavropoulou E., Skoufos I., Tzora A., Alexopoulos A., Bezirtzoglou E. (2018). Biodiversity and microbial resistance of lactobacilli isolated from the traditional Greek cheese kopanisti. Front. Microbiol..

[46] Koutsoukis C., Voidarou C., Demertzis P.G., Akrida-Demertzi K. (2017). Effect of the composition of grazing matter on the quality characteristics of the traditional greek dairy product “Tsalafouti”. J. Environ. Sci. Toxicol. Food Technol..

[47] Marco M.L., Heeney D., Binda S., Cifelli C.J., Cotter P.D., Foligné B., Gänzle M., Kort R., Pasin G., Pihlanto P. (2017). Health benefits of fermented foods: Microbiota and beyond. Curr. Opin. Biotechnol..

[48] Kim D.H., Jeong D., Song K.Y., Seo K.H. (2018). Comparison of traditional and backslopping methods for kefir fermentation based on physicochemical and microbiological characteristics. LWT Food Sci. Technol..

[49] Ashaolu J.T., Reale A. (2020). A holistic review on Euro-Asian lactic acid bacteria fermented cereals and vegetables. Microorganisms.

[50] Anal K.A. (2019). Quality ingredients and safety concerns for traditional fermented foods and beverages from Asia: A review. Fermentation.

[51] Farnworth Ρ.Ε. (2005). Kefir—A complex probiotic. Food Sci. Technol. Bull. Funct. Foods.

[52] Han D.M., Chun B.H., Feng T., Kim H.M., Jeon C.O. (2020). Dynamics of microbial communities and metabolites in ganjang, a traditional Korean fermented soy sauce, during fermentation. Food Microbiol..

[53] Greenwalt C.J., Steinkraus K.H., Ledford R.A. (2000). Kombucha, the fermented tea: Microbiology, composition, and claimed health effects. J. Food Prot..

[54] Terefe S.N. (2016). Food fermentation. Ref. Modul. Food Sci..

[55] Dirar H.A. (1992). Sudan’s Fermented Food Heritage, in Applications of Biotechnology to Traditional Fermented Foods.

[56] Paulová L., Patáková P., Brányik T., Teixeira J., Vincente A.A. (2013). Advanced fermentation processes. Engineering Aspects of Food Biotechnology.

[57] Mishra S.S., Ray C.R., Panda K.S., Montent D. (2017). Technological innovations in processing of fermented foods an overview. Fermented Foods Part II: Technological Intervention.

[58] Tamang J.P., Tamang J.P., Kailasapathy K. (2010). Diversity of fermented foods. Fermented Foods and Beverages of the World.

[59] Bokulich N.A., Bamforth C.W. (2013). The microbiology of malting and brewing. Microbiol. Mol. Biol. Rev..

[60] Chilton N.S., Burton P.J., Reid G. (2015). Inclusion of fermented foods in food guides around the world. Nutrients.

[61] Buckenhiiskes J.H. (1993). Selection criteria for lactic acid bacteria to be used as starter cultures for various food commodities. FEMS Microbiol. Rev..

[62] Mcfeeters R.F. (2004). Fermentation microorganisms and flavor changes in fermented foods. J. Food Sci..

[63] Yim G.D., Jang H.K., Chung Y.K. (2015). Effect of GdL addition on physico-chemical properties of fermented sausages during ripening. Korean J. Food Sci. Anim. Resour..

[64] Srivastava J.R. (2018). Enhanced shelf life with improved food quality from fermentation processes. J. Food Technol. Preserv..

[65] Asioli D., Aschemann-Witzel J., Caputo V., Vecchio R., Annunziata A., Naes T., Varela P. (2017). Making sense of the “clean label” trends: A review of consumer food choice behavior and discussion of industry implications. Food Res. Int..

[66] Mathur H., Beresford P.T., Cotter D.P. (2020). Health benefits of Lactic Acid Bacteria (LAB) fermentates. Nutrients.

[67] Pessione E., Cirrincione S. (2016). Bioactive molecules released in food by lactic acid bacteria: Encrypted peptides and biogenic amines. Front. Microbiol..

[68] Muhialdin J.B., Hassan Z., Sadon K.S. (2011). Biopreservation of food by lactic acid bacteria against spoilage fungi. Ann. Food Sci. Technol..

[69] Hansen E.B. (2002). Commercial bacterial starter cultures for fermented foods of the future. Int. J. Food Microbiol..

[70] Giraffa G. (2004). Studying the dynamics of microbial populations during food fermentation. FEMS Microbiol. Rev..

[71] Franz C.M.A.P., Huch M., Mathara J.M., Abriouel H., Benomar N., Reid G., Holzapfel W.H. (2014). African fermented foods and probiotics. Int. J. Food Microbiol..

[72] Odunfa S.A., Oyewole O.B. (1997). African Fermented Foods.

[73] Olasupo N.A., Odunfa S.A., Obayori O.S., Tamang J.P., Kailasapathy K. (2010). Ethnic African fermented foods. Fermented Foods and Beverages of the World.

[74] Wood B.J.B. (1998). Microbiology of Fermented Foods.

[75] Baschali A., Tsakalidou E., Kyriacou A., Karavasiloglou N., Matalas A.L. (2017). Traditional low-alcoholic and non-alcoholic fermented beverages consumed in European countries: A neglected food group. Nutr. Res. Rev..

[76] Ozer B., Kirmaci H. (2014). Fermented milks: Products of eastern europe and Asia. Encyclopedia of Food Microbiology. Ref. Modul. Food Sci..

[77] Buchanan R., Doyle M.P., Buchanan R.L. (2013). Food Microbiology: Fundamentals and Frontiers.

[78] Cocolin L., Ercolini D. (2008). Molecular Techniques in the Microbial Ecology of Fermented Foods.

[79] Hussain A.M., El Sheikha A.F., Levin R.E., Xu J. (2018). Molecular Techniques for the Identification of LAB in Fermented Cereal and Meat Products. Molecular Techniques in Food Biology: Safety, Biotechnology, Authenticity & Traceability.

[80] El Sheikha A.F., El Sheikha A.F., Levin R.E., Xu J. (2018). Molecular techniques and lactic acid-fermented fruits and vegetables: Why and how?. Molecular Techniques in Food Biology: Safety, Biotechnology, Authenticity & Traceability.

[81] Zheng J., Wittouck S., Salvetti E., Franz C.M., Harris H.M., Mattarelli P., O’Toole P.W., Pot B., Vandamme P., Walter J. (2020). A taxonomic note on the genus *Lactobacillus*: Description of 23 novel genera, emended description of the genus *Lactobacillus Beijerinck* 1901, and union of *Lactobacillaceae* and *Leuconostocaceae*.. Int. J. Syst. Evol. Microbiol..

[82] Diaz M., Kellingray L., Akinyemi N., Adefiranye O.O., Olaonipekun B.A., Bayili G.R., Ibezim J., Du Plessis A.S., Houngbédji M., Kamya D. (2019). Comparison of the microbial composition of African fermented foods using amplicon sequencing. Sci. Rep..

[83] El Sheikha A.F., Hu M.D. (2020). Molecular techniques reveal more secrets of fermented foods. Crit. Rev. Food Sci. Nutr..

[84] Bamforth W.C. (2005). Food, Fermentation and Microorganisms.

[85] Tamang J.P., Tamang B., Schillinger U., Franz C.M.A.P., Gores M., Holzapfel W.H. (2005). Identification of predominant lactic acid bacteria isolated from traditional fermented vegetable products of the Eastern Himalayas. Int. J. Food Microbiol..

[86] Tamang B., Tamang J.P., Schillinger U., Franz C.M.A.P., Gores M., Holzapfel W.H. (2008). Phenotypic and genotypic identification of lactic acid bacteria isolated from ethnic fermented tender bamboo shoots of North East India. Int. J. Food Microbiol..

[87] Bourdichon F., Casaregola S., Farrokh C., Frisvad C.J., Gerds L.M. (2012). Food fermentations: Microorganisms with technological beneficial use. Int. J. Food Microbiol..

[88] Tamang J.P. (2015). Naturally fermented ethnic soybean foods of India. J. Ethn. Foods.

[89] Tamang J.P., Fleet G.H., Satyanarayana T., Kunze G. (2009). Yeasts diversity in fermented foods and beverages. Yeasts biotechnology: Diversity and Applications.

[90] Mandhania M.H., Paul D., Suryavanshi M.V., Sharma L., Chowdhury S., Diwanay S.S., Diwanay S.S., Shouche Y.S., Patole M.S. (2019). Diversity and succession of microbiota during fermentation of the traditional Indian food idli. Appl. Environ. Microbiol..

[91] Haruta S., Ueno S., Egawa I., Hashiguchi K., Fujii A., Nagano M., Ishii M., Igarashi Y. (2006). Succession of bacterial and fungal communities during a traditional pot fermentation of rice vinegar assessed by PCR-mediated denaturing gradient gel electrophoresis. Int. J. Food Microbiol..

[92] Sengun Y.I., Karabiyikli S. (2011). Importance of acetic acid bacteria in food industry. Food Control.

[93] Hinrichsen L.L., Montel M.B., Talon R. (1994). Proteolytic and lipolytic activities of Micrococcus roseus (65), Halomonas elongate (16) and Vibrio spp. (168) isolated from Danish bacon curing brines. Int. J. Food Microbiol..

[94] Papalexandratou Z., Vrancken G., De Bruyne K., Vandamme P., De Vuyst L. (2011). Spontaneous organic cocoa bean box fermentations in Brazil are characterized by a restricted species diversity of lactic acid bacteria and acetic acid bacteria. Food Microbiol..

[95] Rogers P.L., Goodman A.E., Heyes R.H. (1984). Zymomonasethanol fermentations. Microbiol. Sci..

[96] Escalante A., Giles-Gomez M., Hernandez G., Cordova-Aguilar M.S., Lopez-Munguia A., Gosset G., Bolivar F. (2008). Analysis of bacterial community during the fermentation of pulque, a traditional Mexican alcoholic beverage, using a polyphasic approach. Int. J. Food Microbiol..

[97] Schubert K., Ludwig W., Springer N., Kroppenstedt R.M., Accolas J.P., Fiedler F. (1996). Two coryneform bacteriaisolated from the surface of French Gruyère and Beaufort cheeses are new species of the genus *Brachybacterium alimentarium* sp. nov. and *Brachybacteriumtyrophermentas* sp. nov. Int. J. Syst. Bacteriol..

[98] Bockelmann W., Willems K.P., Neve H., Heller K.H. (2005). Cultures for the ripening of smear cheeses. Int. Dairy J..

[99] Leclercq-Perlat M.N., Corrieu G., Spinnler H.E. (2007). Controlled production of Camembert-type cheeses: Part III role of the ripening microflora on free fatty acid concentrations. J. Dairy Res..

[100] Bonnarme P., Lapadatescu C., Yvon M., Spinnler H.E. (2001). L-methionine degradation potentialities of cheese-ripening microorganisms. J. Dairy Res..

[101] O’Mahony T., Rekhif N., Cavadini C., Fitzgerald G.F. (2001). The application of a fermented food ingredient containing ‘variacin’, a novel antimicrobial produced by *Kocuriavarians*, to control the growth of *Bacillus cereus* in chilled dairy products. J. Appl. Microbiol..

[102] Neffe-Skocińsk K., Wójciak K., Zielińska D., Rao V., Rao L.G. (2016). Probiotic Microorganisms in Dry Fermented Meat Products.

[103] Xiao J.Z., Takahashi S., Nishimoto M., Odamaki T., Yaeshima T., Iwatsuki K., Kitaoka M. (2010). Distribution of in vitro fermentation ability of lacto-N-biose I, a major building block of human milk oligosaccharides, in bifidobacterial! strains. Appl. Environ. Microbiol..

[104] Kubo Y., Rooney A.P., Tsukakoshi Y., Nakagawa R., Hasegawa H., Kimura K. (2011). Phylogenetic analysis of Bacillus subtilis strains applicable to natto (fermented soybean) production. Appl. Environ. Microbiol..

[105] Moreno F.M.R., Sarantinopoulos P., Tsakalidou E., De Vuyst L. (2006). The role and application of enterococci in food and health. Int. J. Food Microbiol..

[106] Jeong D.W., Heo S., Lee J.H. (2017). Safety assessment of *Tetragenococcushalophilus* isolates from doenjang, a Korean high-salt-fermented soybean paste. Food Microbiol..

[107] Thokchom S., Joshi S.R. (2013). Physicochemical Analysis of Ethnically Fermented Soybean Products of North-East India and Molecular Characterization of Associated Lactic Acid Bacteria. Proc. Natl. Acad. Sci. India Sect. B Biol. Sci..

[108] Collins M.D., Samelis J., Metaxopoulos J., Wallbanks S. (1993). Taxonomic studies on some leuconostoc-like organisms from fermented sausages: Description of a new genus Weissellafor the Leuconostocparamesenteroides group of species. J. Appl. Bacteriol..

[109] Romero J., Ilabaca C., Ruiz M., Jara C. (2018). *Oenococcusoeni* in chilean red wines: Technological and genomic characterization. Front. Microbiol..

[110] Nychas G., Arkoudelos J.S. (1990). Microbiological and physicochemical changes in minced meats under carbon dioxide, nitrogen or air at 3 °C. Int. J. Food Sci. Technol..

[111] Irlinger F. (2008). Safety assessment of dairy microorganisms: Coagulase-negative staphylococci. Int. J. Food Microbiol..

[112] Ouadghiri M., Amar M., Vancanneyt M., Swings J. (2005). Biodiversity of lactic acid bacteria in Moroccan soft white cheese (Jben). FEMS Microbiol. Lett..

[113] Georgalaki M.D., Sarantinopoulos P., Ferreira E.S., De Vuyst L., Kalantzopoulos G., Tsakalidou E. (2000). Biochemical properties of Streptococcus macedonicusstrains isolated from Greek Kasseri cheese. J. Appl. Microbiol..

[114] Dobson C.M., Deneer H., Lee S., Hemmingsen S., Glaze S., Ziola B. (2002). Phylogenetic analysis of the genus *Pediococcus*, including *Pediococcusclaussenii* sp. nov., a novel lactic acid bacterium isolated from beer. Int. J. Syst. Evol. Microbiol..

[115] Shukla R., Goyal A. (2014). Probiotic potential of pediococcuspentosaceus CRAG3: A new isolate from fermented cucumber. Probiotics Antimicrob Proteins.

[116] Anumpa A., Tamang P.J. (2020). Diversity of filamentous fungi isolated from some amylase and alcohol-producing starters of India. Front. Microbiol..

[117] Tamang J.P., Samuel D., Tamang J.P., Kailasapathy K. (2010). Dietary culture and antiquity of fermented foods and beverages. Fermented Foods and Beverages of the World.

[118] Lee S.C., Billmyre R.B., Li A., Carson S., Sykes S.M., Huh E.Y., Mieczkowski P., Ko D.C., Cuomo C.A., Heitman J. (2014). Analysis of a food-borne fungal pathogen outbreak: Virulence and genome of a *Mucor circinelloides* isolate from yogurt. MBio.

[119] Thapa S., Tamang J.P. (2004). Product characterization of kodo ko jaanr: Fermented finger millet beverage of the Himalayas. Food Microbiol..

[120] Suesse A.R., Norton G.A., van Leeuwen J. (2016). Pilot-scale continuous-flow hydrothermal liquefaction of filamentous fungi. Energy Fuels.

[121] Chen B., Wu Q., Xu Y. (2014). Filamentous fungal diversity and community structure associated with the solid-state fermentation of Chinese Maotai-flavor liquor. Int. J. Food Microbiol..

[122] Rico R.O.G., Chavez R., Fierro F., Laich F., Leitão A.L. (2011). Mold-fermented foods: *Penicillium* spp. as ripening agents in the elaboration of cheese and meat products. Mycofactories.

[123] Gripon J.C., Spinnler H.E. (2004). Surface Mould-Ripened Cheeses. Cheese Chem. Phys. Microbiol..

[124] Nout R., Samson R.A., Hoekstra E.S., Frisvad J.C., Filtenborg O. (2000). Useful role of fungi in food processing. Introduction to Food- and Airborne Fungi.

[125] Nout R., Dijksterhuis J., Samson R.A. (2007). The colonizing fungus as a food provider. Food Mycology. A Multifaceted Approach to Fungi and Food.

[126] Mogensen J.M., Varga J., Thrane U., Frisvad J.C. (2009). Aspergillus acidusfrom Puerh tea and black tea does not produce ochratoxin A and fumonisin B2. Int. J. Food Microbiol..

[127] Tsang C.C., Tang J.Y.M., Lou S.K., Woo C.Y.P. (2018). Taxonomy and evolution of Aspergillus, Penicillium and Talaromyces in the omics era—Past, present and future. Comput. Struct. Biotechnol. J..

[128] Adekoya I., Obadina A., Phoku J., Nwinyi O., Njobeh P. (2007). Contamination of fermented foods in Nigeria with fungi. LWT Food Sci. Technol..

[129] Adebo O.A., Kayitesi E., Njobeh P.B. (2019). Reduction of mycotoxins during fermentation of whole grain sorghum to whole grain ting (a Southern African Food). Toxins.

[130] Karlovsky P., Suman M., Berthiller F., De Meester J., Eisenbrand G., Perrin I., Oswald I.P., Speijers G., Chiodini A., Recker T. (2016). Impact of food processing and detoxification treatments on mycotoxin contamination. Mycotoxin Res..

[131] Shakeel Q., Lyu A., Zhang J., Wu M., Li G., Hsiang T., Yang L. (2018). Biocontrol of *Aspergillus flavus* on peanut kernels using Streptomyces yanglinensis 3–10. Front. Microbiol..

[132] Bachmann H.P., Bobst C., Butikofer U., Casey M.G., Dalla Torre M., Frohlich-Wyder M.T., Furst M. (2005). Occurrence and significance of Fusarium domesticumalias Anticollantion smear-ripened cheeses. LWT Food Sci. Technol..

[133] Grafenham T.Y., Schroers H.-J., Nirenberg H.I., Seifert K.A. (2011). An overview of the taxonomy, phylogeny, and typification of nectriaceous fungi in Cosmospora, Acre-monium, Fusarium, Stilbella, and Volutella. Stud. Mycol..

[134] Thrane U., Dijksterhuis J., Samson R.A. (2007). Fungal protein for food. Food Mycology. A Multifaceted Approach to Fungi and Food.

[135] Gryganskyi P.A., Golan J., Dolatabadi S., Mondo S., Robb S., Idnurm A., Muszewska A., Steczkiewicz K., Masonjones S., Liao H.-L. (2018). Phylogenetic and phylogenomic definition of *Rhizopus* species. G3.

[136] Thanh N.V., Mai T.L., Duong A.T. (2008). Microbial diversity of traditional Vietnamese alcohol fermentation starters (banh men) as determined by PCR-mediated DGGE. Int. J. Food Microbiol..

[137] Ly S., Mith H., Tarayre C., Taminiau B., Daube G., Fauconnier M.L., Delvigne F. (2018). Impact of microbial composition of Cambodian traditional dried starters (Dombea) on flavor compounds of rice wine: Combining amplicon sequencing with HP-SPME-GCMS. Front. Microbiol..

[138] Lv X.C., Huang Z.Q., Zhang W., Rao P.F., Ni L. (2012). Identification and characterization of filamentous fungi isolated from fermentation starters for Hong Qu glutinous rice wine brewing. J. Gen. Appl. Microbiol..

[139] Londoño-Hernández L., Ramírez-Toro C., Ruiz H.A., Ascacio-Valdés J.A., Aguilar-Gonzalez M.A., Rodríguez-Herrera R., Aguilar C.N. (2017). Rhizopus oryzae—Ancient microbial resource with importance in modern food industry. Int. J. Food Microbiol..

[140] Bora S.S., Keot J., Das S., Sarma K., Barooah M. (2016). Metagenomics analysis of microbial communities associated with a traditional rice wine starter culture (Xaj-pitha) of Assam. India. 3 Biotech.

[141] Pitt J.I., Cruickshank R.H., Leistner L.O. (1986). Penicillium commune, P. camembertii, the origin of white cheese moulds, and the production of cyclopiazonic acid. Food Microbiol..

[142] Polonelli L., Morace G., Rosa R., Castagnola M., Frisvad J.C. (1987). Antigenic characterisation of Penicillium camembertiand related common cheese contaminants. Appl. Environ. Microbiol..

[143] Giraud F., Giraud T., Aguileta G., Fournier E., Samson R., Cruaud C., Lacoste S., Ropars J., Tellier A., Dupont J. (2010). Microsatellite loci to recognize species for the cheese starter and contaminating strains associated with cheese manufacturing. Int. J. Food Microbiol..

[144] Ropars J., Villavicencio L.M., Snirc A., Lacoste S., Giraud T. (2017). Blue cheese-making has shaped the population genetic structure of the mould *Penicillium roqueforti*.. PLoS ONE.

[145] Vallone L., Giardini A., Soncini G. (2014). Secondary metabolites from *Penicillium roqueforti*, a starter for the production of gorgonzola cheese. Ital. J. Food Saf..

[146] Hymery N., Vasseur V., Coton M., Mounier J., Jany J.L., Barbier G., Coton E. (2014). Filamentous fungi and mycotoxins in cheese: A review. Compr. Rev. Food Sci. Food Saf..

[147] Siemens K., Zawistowski J. (1993). Occurrence of PR imine, a metabolite of Penicillium roqueforti, in blue cheese. J. Food Prot..

[148] Engel G., Prokopek D. (1979). KeinNachweis von Penicillium roqueforti-Toxin in Kase. Milschwissenschaft.

[149] Engel G., Prokopek D. (1980). KeinNachweis von *Patulin* and *Penicillinsaure* in mit Patulin- und Penicillinsaure-bildenden Penicillium roqueforti-Stammenhergestell-ten Kasen. Milchwissenschaft.

[150] Nandy K.S., Srivastava R.K. (2018). A review on sustainable yeast biotechnological processes and applications. Microbiol. Res..

[151] Kurtzman C., Fell W.J., Boekhout T., Robert V., Cletus Kurtzman J.W., Boekhout F.T. (2011). Methods for isolation, phenotypic characterization and maintenance of yeasts. The Yeasts.

[152] Boekhout T., Robert V. (2003). Yeasts of the World—Morphology, physiology, sequences and identification by T.; Boekhout, V.; Robert, M. Th. Smith, J.; Stalpers, D.; Yarrow, P.; Boer, R.; Buis, G.; Gijswijt, C.P.; Kurtzman, J.W.; Fell, E.; Guého, J. Guillot, and I. Roberts (2002) ISBN 90-75000-47-2 (CD-ROM). ETI Information Services Ltd. UNESCO. Mycologist.

[153] Coton E., Coton M., Levert D., Casaregola S., Sohier D. (2006). Yeast ecology in French cider and black olive natural fermentations. Int. J. Food Microbiol..

[154] Hurtado A., Reguant C., Esteve-Zarzoso B., Bordons A., Rozès N. (2008). Microbial population dynamics during the processing of Arbequina table olives. Food Res. Int..

[155] Arroyo-López F.N., Durán-Quintana M.C., Ruiz-Barba J.L., Querol A., Garrido-Fernández A. (2006). Use of molecular methods for the identification of yeast associated with table olives. Food Microbiol..

[156] Nychas G.J., Panagou E.Z., Parker M.L., Waldron K.W., Tassou C.C. (2002). Microbial colonization of naturally black olives during fermentation and associated biochemical activities in the cover brine. Lett. Appl. Microbiol..

[157] Sánchez A.H., García P., Rejano L. (2006). Elaboration of table olives. Grasas y Aceites.

[158] Vrancken G., De Vuyst L., Van der Meulen R., Huys G., Vandamme P., Daniel H.-M. (2010). Yeast species composition differs between artisan bakery and spontaneous laboratory sourdoughs. FEMS Yeast Res..

[159] Bhalla T.C., Tulasi S., Gotthard K. (2017). Yeasts and Traditional Fermented Foods and Beverages. Yeast Diversity in Human Welfare.

[160] Ahmed Z., Wang Y., Ahmad A., Khan S.T., Nisa M., Ahmad H., Afreen A. (2013). Kefir and health: A contemporary perspective. Crit. Rev. Food Sci. Nutr..

[161] Banjara N., Suhr J.M., Hallen-Adams E.E. (2015). Diversity of yeast and mold species from a variety of cheese types. Curr. Microbiol..

[162] Suharja A.S., Henriksson A., Liu S.Q. (2012). Impact of saccharomyces cerevisiae on viability of probiotic lactobacillus rhamnosus in fermented milk under ambient conditions. J. Food Process. Preserv..

[163] Adenye B.J., Laleye S.A., Akinduro H.A. (2006). Spoilage of some stored fermented foods in Southeastern Nigeria. J. Biol. Sci..

[164] Metaxopoulos J., Mataragas M., Drosinos E.H. (2002). Microbial interaction in cooked cured meat products under vacuum or modified atmosphere at 4 °C. J. Appl. Microbiol..

[165] Singh V.P., Pathak V., Verma A.K. (2002). Fermented meat products: Organoleptic qualities and biogenic amines. A review. Am. J. Food Technol..

[166] Eurostat Statistics Explained, ec.europa.eu 24-10-2020. Milk and Milk Product Statistics. https://ec.europa.eu/eurostat/statistics-explained/index.php/Milk_and_milk_product_statistics.

[167] Rul F., Ray R.C., Montet D. (2017). Yogurt: Microbiology, organoleptic properties and probiotic potential. Fermented Food—Part II: Technological Interventions.

[168] Corsetti A., Settanni L. (2007). Lactobacilli in sourdough fermentation. Food Res. Int..

[169] Blandino A., Al-Aseeri M.E., Pandiella S.S., Cantero D., Webb C. (2003). Cereal-based fermented foods and beverages. Food Res. Int..

[170] De Vuyst L., Vrancken G., Ravyts F., Rimaux T., Weckx S. (2009). Biodiversity, ecological determinants, and metabolic exploitation of sourdough microbiota. Food Microbiol..

[171] Yonzan H., Tamang J.P. (2013). Optimization of traditional processing of *Selroti,* a popular cereal based fermented food. J. Sci. Ind. Res..

[172] Sridevi J., Halami P.M., Vijayendra S.V.N. (2010). Selection of starter cultures for idli batter fermentation and their effect on quality of idli. J. Food Sci. Technol..

[173] Soni S.K., Sandhu D.K., Vilkhu K.S., Kamra N. (1986). Microbiological studies on dosa fermentation. Food Microbiol..

[174] Hamad S.H., Dieng M.M.C., Ehrmann M.A., Vogel R.F. (1997). Characterisation of the bacterial flora of Sudanese sorghum flour and sorghum sourdough. J. Appl. Microbiol..

[175] Gomes R.J., Borges M.F., Rosa M.F., Castro-Gómez R.J.H., Spinosa W.A. (2018). Acetic Acid Bacteria in the Food Industry: Systematics, Characteristics and Applications. Food Technol. Biotechnol..

[176] Sengun I.Y., Nielsen D.S., Karapinar M., Jakobsen M. (2009). Identification of lactic acid bacteria isolated from Tarhana, a traditional Turkish fermented food. Int. J. Food Microbiol..

[177] De Vuyst L., Neysens P. (2005). The sourdough microflora: Biodiversity and metabolic interactions. Trends Food Sci. Technol..

[178] Reese A., Madden A.A., Joossens M., Lacaze G., Dunn R.R. (2020). Influences of ingredients and bakers on the bacteria and fungi in sourdough starters and bread. mSphere.

[179] Minervini F., Lattanzi A., De Angelis M., Di Cagno R., Gobbetti M. (2012). Influence of artisan bakery- or laboratory-propagated sourdoughs onthe diversity of lactic acid bacterium and yeast microbiotas. Appl. Environ. Microbiol..

[180] Ercolini D., Pontonio E., De Filippis F., Minervini F., La Storia A., Gobbetti M., Di Cagno R. (2013). Microbial ecology dynamics during rye and wheat sourdough preparation. Appl. Environ. Microbiol..

[181] Pulvirenti A., Solieri L., Gullo M., Vero L.D., Giudici P. (2004). Occurrence anddominance of yeast species in sourdough. Lett. Appl. Microbiol..

[182] De Vuyst L., Van Kerrebroeck S., Harth H., Huys G., Daniel H.M., Weckx S. (2014). Microbial ecology of sourdough fermentations: Diverse or uniform?. Food Microbiol..

[183] Hammes W.P., Brandt M.J., Francis K.L., Rosenheim J., Seitter M.F.H., Vogelmann S.A. (2004). Microbial ecology of cereal fermentations. Trends Food Sci. Technol..

[184] Foschino R., Arrigoni C., Picozzi C., Mora D., Galli A. (2001). Phenotypic and genotypic aspects of Lactobacillus sanfranciscensis strains isolated from sourdoughs in Italy. Food Microbiol..

[185] Foschino R., Terraneo R., Mora D., Galli A. (1999). Microbial characterization of sourdoughs for sweet baked products. Ital. J. Food Sci..

[186] Foschino R., Gallina S., Andrighetto C., Rossetti L., Galli A. (2004). Comparison of cultural methods for the identification and molecular investigation of yeasts from sourdoughs for Italian sweet baked products. FEMS Yeast Res..

[187] Moroni A., Arendt K.E., Bello D.F. (2011). Biodiversity of lactic acid bacteria and yeasts in spontaneously-fermented buckwheat and teff sourdoughs. Food Microbiol..

[188] Russo P., Fares C., Longo A., Soano G., Capozzi V. (2017). *Lactobacillus plantarum* with broad antifungal activity as a protective starter culture for bread production. Foods.

[189] Kara Ali M., Outili N., Ait Kaki A., Cherfia R., Benhassine S., Benaissa A., KacemChaouche N. (2017). Optimization of baker’s yeast production on date extract using Response Surface Methodology (RSM). Foods.

[190] Kaur P., Ghoshal G., Banerjee U.C. (2019). Traditional bio-preservation in beverages: Fermented beverages. Preservatives and preservation approaches in beverages. Sci. Beverages.

[191] Altay F., Karbancıoglu-Güler F., Daskaya-Dikmen C., Heperkan D. (2013). A review on traditional Turkish fermented non-alcoholic beverages: Microbiota, fermentation process and quality characteristics. Int. J. Food Microbiol..

[192] Varzakas T., Zakynthinos G., Proestos C., Radwanska M., Yildiz F., Wiley R.C. (2017). Fermented vegetables. Minimally Processed and Refrigerated Fruits and Vegetables.

[193] Zabat M.A., Sano W.H., Wurster J.I., Cabral D.J., Belenky P. (2018). Microbial community analysis of sauerkraut fermentation reveals a stable and rapidly established community. Foods.

[194] Bell V., Ferrão J., Pimentel L., Pintado M., Fernandes T. (2018). One health, fermented foods, and gut microbiota. Foods.

[195] Bell V., Ferrão J., Fernandes T. (2017). Nutritional guidelines and fermented food frameworks. Foods.

[196] Banan-MwineDaliri E., Lee B.H. (2015). New perspectives on probiotics in health and disease. Food Sci. Hum. Wellness..

[197] Dewulf E., Cani P., Claus S., Fuentes S., Puylaert P., Neyrinck A., Bindels L., de Vos W., Gibson G., Thissen J.-P. (2013). Insight into the prebiotic concept: Lessons from an exploratory, double blind intervention study with inulin-type fructans in obese women. Gut.

[198] Lee K., Lee H., Cho H., Kim Y. (2005). Role of the conjugated linoleic acid in the prevention of cancer. Crit. Rev. Food Sci. Nutr..

[199] Lyte M. (2011). Probiotics function mechanistically as delivery vehicles for neuroactive compounds: Microbial endocrinology in the design and use of probiotics. Bioessays.

[200] Hsiao E., McBride S., Hsien S., Sharon G., Hyde E., McCue T., Codelli J., Chow J., Reisman S., Petrosino J. (2013). Microbiota modulate behavioral and physiological abnormalities associated with neurodevelopmental disorders. Cell.

[201] Greer R., Morgun A., Shulzhenko N. (2013). Bridging immunity and lipid metabolism by gut microbiota. J. Allergy Clin. Immunol..

[202] Antoniadou M., Varzakas T. (2020). Breaking the vicious circle of diet, malnutrition and oral health for the independent elderly. Crit. Rev. Food Sci. Nutr..

[203] Chen T., Yu W.H., Izard J. (2010). The human oral microbiome database: A web accessible resource for investigating oral microbe taxonomic and genomic information. Database.

[204] Chopra R., Mathur S. (2014). Probiotics in dentistry: A boon or sham. Dent. Res. J..

[205] Stamatova I., Meurman J.H. (2009). Probiotics: Health benefits in the mouth. Am. J. Dent..

[206] Antoniadou M., Varzakas T., el Enshasy H., Yang S.T. (2020). Probiotics and prebiotics and their effect in food and human health. New perspectives. Probiotics the Natural Microbiota in Living Organisms.

[207] Gungor O.E., Kirzioglu Z., Kivanc M. (2015). Probiotics: Can they be used to improve oral health?. Benef. Microbes.

[208] Mahasneh S.A., Mahasneh A.M. (2017). Probiotics: A promising role in dental health. Dental J..

[209] Beighton D. (2005). The complex oral microflora of high-risk individuals and groups and its role in the caries process. Community Dental Oral Epidemiol..

[210] Falenski A., Mayer-Scholl A., Filter M., Göllner C., Appel B., Nöckler K. (2011). Survival of *Brucella* spp. in mineral water, milk and yogurt.. Int. J. Food Microbiol..

[211] Pazakova J., Turek P., Laciakova A. (1997). The survival of Staphylococcus aureus during the fermentation and storage of yoghurt. J. Appl. Microbiol..

[212] Dobson A., Cotter D.P., Ross R.P., Hill G. (2012). Bacteriocin production: A probiotic trait?. Appl. Environ. Microbiol..

[213] Yang E., Fan L., Jiang Y., Doucette C., Fillmore S. (2012). Antimicrobial activity of bacteriocin-producing lactic acid bacteria isolated from cheeses and yogurts. AMB Express.

[214] Cotter P.D., Hill C., Ross R.P. (2005). Bacteriocins: Developing innate immunity for food. Nat. Rev. Microbiol..

[215] Barbosa A.A.T., Mantovani H.C., Jain S. (2017). Bacteriocins from lactic acid bacteria and their potential in the preservation of fruit products. Crit. Rev. Biotechnol..

[216] Voidarou C., Alexopoulos A., Tsinas A., Rozos G., Tzora A., Skoufos I., Varzakas T., Bezirtzoglou E. (2020). Effectiveness of bacteriocin-producing Lactic acid bacteria and *Bifidobacterium* isolated from honeycombs against spoilage microorganisms and pathogens isolated from fruits and vegetables. Appl. Sci..

[217] Borges S., Teixeira P. (2016). Application of bacteriocins in food and health care. Bacteriocins: Production, Applications and Safety.

[218] https://www.who.int/news-room/fact-sheets/detail/antimicrobial-resistance.

[219] Caniça M., Manageiro V., Abriouel H., Moran-Gilad J., Franz C.M. (2019). Antibiotic resistance in foodborne bacteria. Trends Food Sci. Technol..

[220] https://www.efsa.europa.eu/en/news/antimicrobial-resistance-eu-infections-foodborne-bacteria-becoming-harder-treat.

[221] https://www.cdc.gov/drugresistance/biggest-threats.html.

[222] Sørum H., L’Abee-Lund M.T. (2002). Antibiotic resistance in food-related bacteria--a result of interfering with the global web of bacterial genetics. Int. J. Food Microbiol..

[223] Davies J., Davies D. (2010). Origins and evolution of antibiotic resistance. Microbiol. Mol. Biol. Rev..

[224] Verraes C., Van Boxstael S., Van Meervenne E., Van Coillie E., Butaye P., Catry B., De Schaetzen M.A., Van Huffel X., Imberechts H., Dierick K. (2013). Antimicrobial resistance in the food chain: A review. Int. J. Environ. Res. Public Health.

[225] MacGowan A., Macnaughton E. (2017). Antibiotic resistance. Medicine.

[226] Anal A.K., Perpetuini G., Petchkongkaew A., Tan R., Avallone S., Tofalo R., Van Nguyen H., Chu-Ky S., Ho P.H., Phan T.T. (2020). Food safety risks in traditional fermented food from South-East Asia. Food Control.

[227] Abriouel H., Knapp C.W., Gálvez A., Benomar N., Frías J., Martínez-Villaluenga C., Peñas E. (2017). Chapter 29—Antibiotic resistance profile of microbes from traditional fermented foods. Fermented Foods in Health and Disease Prevention.

[228] Maidana D.S., Ficoseco S.A., Bassi D., Cocconcelli P.S. (2020). Biodiversity and technological-functional potential of lactic acid bacteria isolated from spontaneously fermented chia sourdough. Int. J. Food Microbiol..

[229] Zarzecka U., Zadernowska A., Chajęcka-Wierzchowska W. (2020). Starter cultures as a reservoir of antibiotic resistant microorganisms. LWT.

[230] Toscano M., Peroni D., De Vecchi E., Mattina R., Drago L. (2013). Microbiological assessment of some powdered infant formulas: From quality to antibiotic resistance evaluation. J. Food Process. Technol..

[231] https://www.efsa.europa.eu/sites/default/files/consultation/120323.pdf.

